# Research priorities to address the global burden of chronic obstructive pulmonary disease (COPD) in the next decade

**DOI:** 10.7189/jogh.11.15003

**Published:** 2021-10-09

**Authors:** Davies Adeloye, Dhiraj Agarwal, Peter J Barnes, Marcel Bonay, Job F van Boven, Jamie Bryant, Gaetano Caramori, David Dockrell, Anthony D'Urzo, Magnus Ekström, Gregory Erhabor, Cristóbal Esteban, Catherine M Greene, John Hurst, Sanjay Juvekar, Ee Ming Khoo, Fanny W Ko, Brian Lipworth, Jose L López-Campos, Matthew Maddocks, David M Mannino, Fernando J Martinez, Miguel A Martinez-Garcia, Renae J McNamara, Marc Miravitlles, Hilary Pinnock, Alison Pooler, Jennifer K Quint, Peter Schwarz, George M Slavich, Peige Song, Andrew Tai, Henrik Watz, Jadwiga A Wedzicha, Michelle C Williams, Harry Campbell, Aziz Sheikh, Igor Rudan

**Affiliations:** 1 Usher Institute, University of Edinburgh, Edinburgh, UK; 2 Vadu Rural Health Program, KEM Hospital Research Centre, Pune, India; 3 Imperial College London, UK; 4 University of Versailles, Versailles, France; 5 University of Groningen, University Medical Center Groningen, Groningen Research Institute for Asthma and COPD (GRIAC), Department of Clinical Pharmacy & Pharmacology, Groningen, the Netherlands; 6 University of Newcastle, Newcastle, New South Wales, Australia; 7 University of Messina, Messina, Italy; 8 Centre for Inflammation Research, University of Edinburgh, Edinburgh, UK; 9 University of Toronto, Toronto, Ontario, Canada; 10 Lund University, Lund, Sweden; 11 Obafemi Awolowo University, Ile-Ife, Nigeria; 12 Galdakao-Usansolo Hospital, Galdakao, Bizkaia, Spain; 13 Royal College of Surgeons in Ireland, Dublin, Ireland; 14 UCL Respiratory, University College London, UK; 15 Faculty of Medicine, University of Malaya, Kuala Lumpur, Malaysia; 16 The Chinese University of Hong Kong, Hong Kong; 17 Scottish Centre for Respiratory Research, University of Dundee, Dundee, UK; 18 Unidad Médico-Quirúrgica de Enfermedades Respiratorias, Instituto de Biomedicina de Sevilla (IBiS); Hospital Universitario Virgen del Rocio – Universidad de Sevilla – CIBERES, Spain; 19 Kings College London, UK; 20 University of Kentucky, Lexington, Kentucky, USA; 21 Weill Cornell Medicine, New York City, New York, USA; 22 Hospital Universitario y Politécnico de La Fe, Valencia, Spain; 23 The University of Sydney, New South Wales, Australia; 24 Pneumology Department, University Hospital Vall d'Hebron and Vall d’Hebron Institut de Recerca (VHIR), Barcelona, Spain; 25 Keele University, Keele, UK; 26 Bone-metabolic Research Unit, Copenhagen University Hospital Rigshospitalet, Denmark; 27 Cousins Center for Psychoneuroimmunology and Department of Psychiatry and Biobehavioral Sciences, University of California, Los Angeles, California, USA; 28 School of Public Health, Zhejiang University School of Medicine, Hangzhou, China; 29 Women's and Children's Hospital, Adelaide, South Australia, Australia; 30 Pulmonary Research Institute at Lungen Clinic Grosshansdorf, Airway Research Center North (ARCN), German Centre for Lung Research (DZL), Germany; 31 Centre for Cardiovascular Science, The Queen's Medical Research Institute, University of Edinburgh, Edinburgh, UK

## Abstract

**Background:**

The global prevalence of chronic obstructive pulmonary disease (COPD) has increased markedly in recent decades. Given the scarcity of resources available to address global health challenges and respiratory medicine being relatively under-invested in, it is important to define research priorities for COPD globally. In this paper, we aim to identify a ranked set of COPD research priorities that need to be addressed in the next 10 years to substantially reduce the global impact of COPD.

**Methods:**

We adapted the Child Health and Nutrition Research Initiative (CHNRI) methodology to identify global COPD research priorities.

**Results:**

62 experts contributed 230 research ideas, which were scored by 34 researchers according to six pre-defined criteria: answerability, effectiveness, feasibility, deliverability, burden reduction, and equity. The top-ranked research priority was the need for new effective strategies to support smoking cessation. Of the top 20 overall research priorities, six were focused on feasible and cost-effective pulmonary rehabilitation delivery and access, particularly in primary/community care and low-resource settings. Three of the top 10 overall priorities called for research on improved screening and accurate diagnostic methods for COPD in low-resource primary care settings. Further ideas that drew support involved a better understanding of risk factors for COPD, development of effective training programmes for health workers and physicians in low resource settings, and evaluation of novel interventions to encourage physical activity.

**Conclusions:**

The experts agreed that the most pressing feasible research questions to address in the next decade for COPD reduction were on prevention, diagnosis and rehabilitation of COPD, especially in low resource settings. The largest gains should be expected in low- and middle-income countries (LMIC) settings, as the large majority of COPD deaths occur in those settings. Research priorities identified by this systematic international process should inform and motivate policymakers, funders, and researchers to support and conduct research to reduce the global burden of COPD.

It is estimated that between 300 and 400 million people globally live with chronic obstructive pulmonary disease (COPD) [1,2]. The growing burden of COPD is particularly concerning in low- and middle-income countries (LMICs), due to increasing rates of smoking, household- and ambient air pollution and other exposures, coupled with large and ageing populations [3-5]. Furthermore, the ongoing COVID-19 pandemic highlighted COPD as a condition that predisposes to increased risk of hospitalisation and death [6]. Given the scarcity of resources available for addressing global health challenges and respiratory medicine being relatively underinvested in, it is important to define research priorities for COPD globally [7-9].

COPD now ranks as the fourth leading cause of death, resulting in around three million deaths each year [8,10-12]. Globally, COPD is estimated to result in economic costs of US$2.1 trillion, and at least half of these costs are now in LMICs [13]. Of this total, an estimated US$1.9 trillion are direct costs such as medical care, while US$0.2 trillion are indirect costs such as missed work [14]. These figures are expected to more than double by the year 2030 [13]. Therefore, a coordinated global response is needed to effectively address the burden of COPD and the very considerable challenges it poses. Prioritising research on COPD could help to motivate leaders, researchers and stakeholders. As resources in global health are generally limited, there is a need to agree on research priorities to guide policymakers and funding organisations as they work to advance the COPD research agenda [15,16].

In response to the heightened interest of the international community in non-communicable diseases, several efforts have been successfully conducted in the past decade to identify research priorities in various fields of global health [17-19]. These initiatives have assisted progress because the process of identification of research priorities has informed governments, funding agencies and the private sector on how to prioritise investments in a systematic way. There was a need for a transparent, systematic and replicable prioritisation process that would be perceived as globally representative and fair. It should involve all relevant stakeholders to mount a coordinated international response to the existing complex and sizable challenges in the field of COPD research. The Child Health and Nutrition Research Initiative (CHNRI) method, now the most commonly used methodological approach to generate research priorities for medicine and health care, was developed to respond to this need for methodological rigour. It has been successfully used as a tool to assist decision making and consensus development in child health and nutrition [20-22] and subsequently been extended to numerous other priority-setting exercises [23], including those in research on disability [24], dementia [25], global mental health [26] and medication safety [27].

Past research prioritisation exercises conducted in the field of COPD have been insightful, but have used a variety of approaches and methods that fell short of being global, systematic or replicable outside of the specific context, or were limited in scope [28-34]. In this paper, we used the CHNRI method [20-22]. We sought to identify a ranked set of COPD research priorities that need to be addressed in the next 10 years to substantially reduce the global impact of COPD – on patients, their families and society at large.

## METHODS

### The CHNRI method

This was a priority-setting study that crowdsourced expert opinion in a systematic way. The CHNRI method is a systematic, transparent, and democratic approach to priority setting for research and health interventions based on collective opinion. It employs the principle of crowdsourcing to collate and score research ideas against a pre-defined set of criteria. Based on submitted opinions of a larger number of experts, funders and policymakers are able to view the strengths, weaknesses, and relative ranking of each proposed research idea. While it allows researchers to independently generate and score research questions, it also involves relevant stakeholders, including patients, carers and support groups, at an early stage of the process, ensuring their ownership in the outcomes [35-41]. Previous experiences with more than a 100 conducted CHNRI exercises are summarized in **Box 1**.

Box 1 **Previous CHNRI exercises, search strategy and selection criteria**

The CHNRI method has been used in over 100 studies led by multilateral organisations (eg, World Health Organization (WHO), United Nations Children's Fund (UNICEF)), national governments (eg, China, India, and South Africa), and funders (eg, The Bill and Melinda Gates Foundation) to set research priorities in areas ranging from the reduction of global child mortality, chronic non-communicable diseases or disability to the efficient execution of national health plans [23,35-41]. Previous experiences and statistical simulations found considerable convergence of collective expert opinion leading to stable and replicable results [40].

To conduct this exercise, in September 2019 we ran searches on Web of Science's Core Collection to identify experts (*ie*. first, last or corresponding lead authors of the top 1% most cited research articles) using the keywords “COPD” or “Chronic Obstructive Pulmonary Disease”. Then, in January 2020, to put our findings in context of the wider literature, we ran additional searches on MEDLINE, Embase, Global Health, and CINAHL to identify studies on COPD priority settings published up to December 2019. We used a combination of Medical Subject Headings (MeSH) on the databases as follows: “*pulmonary disease, chronic obstructive/ or *bronchitis, chronic/ or *pulmonary emphysema/” *AND* “*morbidity/ or *mortality/ *risk factors/” AND burden adj3 disease” *AND* “Health Priorit*/ or priority setting*/ or Resource allocation/”. No language or geographical restrictions were applied.

Although we took steps to maximise response rate using a three-stage invitation process (initial contact, invitation to submit ideas, and invitation to submit scores), and a follow-up after four weeks for non-response, the overall response rate can be considered quite low, and it falls in line invitation to participate in a research without incentives, also observed in previous CHNRI exercises [23-25].

### Management group

We established a Management Group affiliated with National Institute of Health Research's RESPIRE Global Health Unit and Centre for Global Health at the Usher Institute, the University of Edinburgh, to identify research priorities to address the global burden of COPD. In September 2019, we developed a protocol to guide this process in line with recently published revised guidelines for the application of the CHNRI method, based on the experience of its use [35-41]. A small Management Group that includes the authors of this report (IR, DA, and AS) coordinated the steps of the priority setting exercise.

### Invitation of experts

We identified 432 experts in COPD research from across the world by searching the Web of Science's Core Collection for the most productive authors in the preceding five-year period, or those who were lead authors (first, last or corresponding) of the top 1% most cited research articles. Search strategies and selection criteria are summarized in **Box 1**. In the first phase of invitation, e-mails were sent to all the experts seeking their participation and with details of the objectives and context of the exercise. In a second e-mail, experts that showed interest were invited to generate a minimum of three research questions. The Management Group then scrutinised the submitted ideas and ensured that the wording of each idea fitted the format for the scoring process. This led to a consolidated list of 230 unique research ideas, that could be categorised in 6 sub-themes (**Box 2**). Then, the experts were re-invited (with a four-week timeline for follow-up) to systematically rank these ideas using pre-agreed criteria, which are listed below.

Box 2 **Research sub-themes**

Six broad research sub-themes were identified:

- Epidemiology and risk factors of COPD (26 research ideas).
- Aetiology and pathophysiology of COPD and exacerbations (43 research ideas).
- Strategies (and policies) for prevention, management, and rehabilitation of COPD (92 research ideas).
- COPD self-management and adherence to treatment (10 research ideas).
- Approaches towards improved diagnosis and classification of COPD (44 research ideas).
- Monitoring disease progression and/or prognosis of COPD (15 research ideas).

### Research context and criteria

The context and the criteria for scoring were defined in line with recommendations from the previous exercises and guidelines [38,41]. The context was defined as “global”, ie, taking into account that the majority of the burden is in LMICs. The timeframe within which the results were expected from proposed research was specified as up to 10 years. The age group of people with COPD was defined as 40 years or older, because the management group agreed that the overwhelming majority of COPD cases would be affecting this age group. The target population include respiratory physicians, researchers, policymakers, funders, patients and support groups across various global settings.

Six independent criteria were agreed by the Management Group and used to discriminate between the many proposed research questions identified:

1. Answerability: Is this research question likely to be answered using the proposed methods and approaches?
2. Effectiveness: Is this research question likely to lead to interventions that will effectively reduce the burden of COPD over time?
3. Feasibility: Is it feasible to address this research question given the existing level of knowledge, capacity and resources?
4. Deliverability: Is this research question likely to lead to interventions or solutions that could be readily implemented and deliverable to population at scale?
5. Burden reduction: Is this research question likely to lead to a significant reduction in COPD burden?
6. Equity: Is this research question likely to reduce inequity in the population?

#### Scoring and analysis

All invited contributors were asked to score each submitted research question using these pre-defined criteria. Experts were offered four response options for scoring: 0 (unlikely to meet the criterion); 0.5 (not sure if it can meet the criterion); 1 (likely to meet the criterion); or left blank if the expert felt insufficiently informed to make a judgment. We generated intermediate scores by calculating the mean of the individual scores for each research question and each criterion received from all experts, and it ranged from 0%-100%. Subsequently, the overall Research Priority Score (RPS) assigned to each research question was a simple mean of all six criteria-specific scores. Average Expert Agreement (AEA), an indicator of the average proportion of scorers that returned the most common answer for a research question was also calculated for each research question to provide an understanding of the level of agreement among scorers. This is expressed as the frequency of the mode (ie, the most common score divided by the total number of scores).


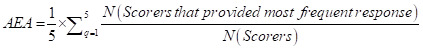


where “*5*” represents the five criteria, and “*N*” is the total number of experts.

## RESULTS

Among the 432 researchers contacted, 64 (14.8%) contributed 264 research questions. We removed the duplicates and consolidated the final list into 230 research questions, which were scored by 34 researchers according to the six criteria of interest. A total of 8 experts from LMICs accepted our invitation and took part in this exercise, although of the initial 432 experts contacted, over 30% were indirectly affiliated or collaborating with researchers in LMICs. Further information on the 64 contributors of research questions and 34 among them who also agreed to take the time and score is provided in the **Online Supplementary Document**, in accordance to General Data Protection Regulation.

### Research priority scores and expert agreement

The overall RPS for the 230 research questions ranged from 0.868 (highest) to 0.243 (lowest). The AEA revealed that across the entire exercise, on average, 43%-82% of scorers provided the same most common answer to a criterion related to the 230 proposed research ideas (Supplementary Online Material). The AEA decreased with decreasing RPS (Spearman's rho (ρ) = 0.7907, *P* <  0.001) suggesting a higher degree of agreement among experts on the top ranked questions and consistent with previous CHNRI findings. See Tables S1-S7 in the **Online Supplementary Document** for RPS and AEA for all 230 research ideas.

### Top-ranked priorities

The top-ranked research priority proposed to develop new effective strategies for smoking cessation. Among the top 20 overall research priorities, six were focused on feasible and cost-effective pulmonary rehabilitation delivery and access, particularly in primary care and low-resource settings. Three of the top 10 overall priorities called for research on improved screening and better diagnostic methods for COPD in primary care and low-resource settings. Further ideas that drew support from scorers involved a better understanding of risk factors for COPD, development of effective training programmes and guidelines for health workers and physicians in low resource settings, and encouraging physical activity (**Table 1**).

**Table 1:** Top 20 research ideas by their overall Research Priority Scores (RPS) and Average Expert Agreement (AEA)

Rank	Research idea	Sub-theme	Answerable?	Effective?	Feasible?	Deliverable?	Impact?	Equity?	Rps	Aea
1	Developing new strategies (including new combinations of pharmacological and non-pharmacological strategies) to improve smoking cessation	III	0.952	0.967	0.790	0.790	0.903	0.806	**0.868**	0.779
2	Identifying feasible and effective modes of delivery of pulmonary rehabilitation in low-resource settings	III	0.935	0.667	0.935	0.903	0.823	0.903	**0.861**	0.779
3	Identifying optimal screening method for COPD in primary care	V	0.938	0.773	0.969	0.924	0.667	0.848	**0.853**	0.814
4	Identifying feasible strategies to improve access to pulmonary rehabilitation for COPD patients whilst retaining cost-effectiveness	III	0.953	0.710	0.953	0.891	0.766	0.813	**0.847**	0.779
5	Identifying approached to scale-up of delivery of effective pulmonary rehabilitation in low resource settings to meet the burden of breathlessness	III	0.900	0.672	0.933	0.850	0.817	0.900	**0.845**	0.730
6	Defining the most affordable, accurate and reliable diagnostic process for respiratory symptoms in low-resource settings based on evidence	V	0.891	0.758	0.823	0.917	0.742	0.935	**0.844**	0.755
7	Exploring the non-smoking risk factors for the development of COPD (eg, premature birth, childhood asthma, genes, biomass fumes exposure, atmospheric pollution, etc.)	I	0.909	0.855	0.844	0.797	0.774	0.875	**0.842**	0.775
8	Identifying optimal diagnostic approach for COPD in low-resource settings	V	0.938	0.742	0.813	0.938	0.703	0.875	**0.835**	0.779
9	Improved understanding of COPD risk factors and their association with COPD incidence and exact effects	I	0.894	0.859	0.984	0.797	0.734	0.734	**0.834**	0.770
10	Identifying optimal approaches to training physicians, health care workers, policy makers and the community in low resource settings about COPD and its risk factors	III	0.894	0.797	0.906	0.813	0.750	0.813	**0.829**	0.779
11	Health policy and systems research on feasibility of establishing pulmonary rehabilitation centers in the communities	III	0.906	0.645	0.969	0.891	0.742	0.781	**0.822**	0.765
12	Identifying strategies that are effective and cost-effective in reducing anxiety and depression among individuals with COPD	III	0.933	0.583	0.919	0.800	0.919	0.742	**0.816**	0.672
13	Developing guidelines for health practitioners in low resource settings to diagnose and treat/manage their COPD patients	V	0.969	0.700	0.875	0.906	0.633	0.766	**0.808**	0.745
14	Identifying the most cost-effective COPD treatment strategies for low resource settings	III	0.903	0.700	0.806	0.806	0.733	0.871	**0.803**	0.725
15	Defining criteria for antibiotics use in acute COPD	III	0.970	0.710	0.938	0.906	0.656	0.594	**0.796**	0.750
16	Developing an eHealth pulmonary rehabilitation program that is as effective as regular rehabilitation in both health benefits and costs	III	0.938	0.661	0.906	0.875	0.703	0.688	**0.795**	0.740
17	Identifying the most effective e-health, m-health and telemedicine interventions for the management of COPD in primary care	III	0.938	0.613	0.906	0.891	0.719	0.688	**0.792**	0.735
18	Identifying feasible and effective means to increase physical activity in patients with newly diagnosed COPD	III	0.919	0.661	0.891	0.839	0.629	0.806	**0.791**	0.711
19	Identifying feasible approaches for providing pulmonary rehabilitation in primary care	III	0.935	0.567	0.903	0.823	0.710	0.774	**0.785**	0.711
20	Studying the effectiveness of physical activity incentive programs on prevention of hospitalizations due to acute exacerbations of COPD	III	0.903	0.694	0.952	0.774	0.625	0.742	**0.782**	0.711

COPD – chronic obstructive pulmonary disease

*Overall Research Priority Score used for the ranking is given in bold.

### Bottom-ranked priorities

Because of very poor scores on one or more of the six criteria, the scorers showed the lowest collective optimism towards ideas that proposed to identify biological pathways that underlie different clinical presentations, then exploring if COPD with airway mucus hypersecretion and higher risk of death should be approached as a separate disease and the basis of day-to-day variability in symptoms. Also, there was low optimism towards highly specific questions that sought to provide answers of limited use and transferability or contribute to refining the existing definitions of COPD and exacerbation. Interestingly, there was not much support for studying gene reprogramming of the epithelium in COPD, piloting replacement of a “COPD” diagnosis with individual clinical and biological phenotyping, evaluating the usefulness of measuring sensory and affective dimension of acute or episodic breathlessness, or evaluating animal models to determine the contributions of a new generation of nicotine products. Finally, the least enthusiasm was shown towards synthesising various lines of evidence to reach a consensus whether COPD is a disease or a disorder (**Table 2**).

**Table 2:** Bottom 20 research ideas (in descending order) by their overall Research Priority Scores (RPS) and Average Expert Agreement (AEA)

RANK	RESEARCH IDEA	SUB-THEME	ANSWERABLE?	EFFECTIVE?	FEASIBLE?	DELIVERABLE?	IMPACT?	EQUITY?	RPS	AEA
230	Synthesizing various lines of evidence to reach a consensus whether COPD is a disease or a disorder	V	0.365	0.111	0.278	0.259	0.111	0.333	**0.243**	0.593
229	Evaluating animal models to determine the contributions of new generation of nicotine products	III	0.556	0.308	0.482	0.315	0.125	0.310	**0.349**	0.525
228	Evaluating the usefulness of measuring sensory and affective dimension of acute or episodic breathlessness (clinical, spirometry, EEG and fMRI) to differentiate breathlessness from panic in COPD	VI	0.556	0.214	0.500	0.414	0.155	0.300	**0.356**	0.554
227	Piloting replacement of diagnosis “COPD” with individual clinical and biological phenotyping	V	0.571	0.293	0.500	0.310	0.172	0.310	**0.360**	0.554
226	Studying gene reprogramming of the epithelium in COPD	II	0.560	0.280	0.558	0.288	0.120	0.357	**0.361**	0.495
225	Studying if adverse effects of beta agonists, taken in the absence of inhaled corticosteroids, are an unrecognised problem in COPD	III	0.692	0.167	0.625	0.393	0.148	0.393	**0.403**	0.554
224	Understanding the basic non-hypoxic pathogenesis of pulmonary hypertension in COPD	II	0.667	0.241	0.607	0.431	0.103	0.379	**0.405**	0.564
223	Studying the pathophysiological processes that lead to emphysema, bronchiectasis and chronic bronchitis - are they the same but just affecting different anatomical sites?	II	0.630	0.321	0.500	0.321	0.286	0.379	**0.406**	0.515
222	Developing new models of care in COPD based on the coming “silver tsunami” and limited resources	III	0.500	0.308	0.407	0.407	0.385	0.500	**0.418**	0.426
221	Studying if chronic non-fully reversible airflow obstruction in never-smokers is really COPD	III	0.630	0.286	0.569	0.339	0.241	0.483	**0.425**	0.515
220	Identifying diagnostic tools to distinguish between the true COPD exacerbation and just a 'bad day'	V	0.569	0.323	0.583	0.552	0.155	0.367	**0.425**	0.544
219	Exploring if measurement of lung stiffness as reactance area (AX) using impulse oscillometry can identify treatment response to LABA/LAMA or triple therapy in patients with GOLD B/D	III	0.793	0.222	0.707	0.464	0.154	0.259	**0.433**	0.569
218	Exploring the basis of day-to-day variability in symptoms (eg, dyspnoea, fatigue, pain, weakness, insomnia, guilt, anxiety, depression, appetite, etc.) in patients with COPD	VI	0.685	0.259	0.625	0.389	0.179	0.483	**0.437**	0.525
217	Exploring if COPD with airway mucus hypersecretion and higher risk of dying should be approached as a separate disease	II	0.565	0.328	0.583	0.533	0.300	0.367	**0.446**	0.525
216	Identifying biological pathways that underlie different clinical presentations	II	0.635	0.310	0.500	0.481	0.250	0.500	**0.446**	0.461
215	Studying the early effects causing a lower maximum lung function at young adulthood and its clinical implications	VI	0.558	0.446	0.625	0.352	0.304	0.429	**0.452**	0.480
214	Identifying drivers of epithelial-mesenchymal transition (EMT) in smoking-related COPD and its relation to the severe risk of COPD and airway cancers	I	0.732	0.389	0.643	0.407	0.241	0.321	**0.456**	0.525
213	Studying value-based COPD care and identifying important values for patients and society to achieve in COPD care (in line with Machteld Huber’s ‘positive health’).	III	0.574	0.370	0.519	0.429	0.224	0.621	**0.456**	0.495
212	Identification of non-coding RNAs that contribute to COPD pathophysiology and development of inhalable ncRNA-based medicines for treatment of COPD symptomology	II	0.712	0.385	0.667	0.389	0.278	0.321	**0.458**	0.510
211	Validating of objective and subjective clinical outcomes specific for disease modifying therapies	III	0.558	0.346	0.589	0.519	0.259	0.500	**0.462**	0.461

COPD – chronic obstructive pulmonary disease, LABA – long-acting β-agonist, LAMA – long-acting muscarinic antagonist; GOLD – Global Initiative for Chronic Obstructive Lung Disease (acronym for guidelines B/D)

*Overall Research Priority Score used for the ranking is given in bold.

### Top-ranked priorities across research criteria

When research ideas were considered by their likelihood of answerability, “Studying whether inhaled corticosteroids increase risk of bacterial infections in COPD” received the maximum score. For likelihood of effectiveness, there was very high agreement that developing new strategies (including new combinations of pharmacological and non-pharmacological strategies) to improve smoking cessation would be the most effective research idea. Based on the likelihood of feasibility, the leading research idea was improved understanding of COPD risk factors and their association with COPD incidence and exact effects, studying whether inhaled corticosteroids increase the risk of bacterial infections in COPD, and identifying optimal screening methods for COPD in primary care. When likelihood of deliverability was analysed, identifying optimal diagnostic approaches for COPD in low-resource settings was ranked as most deliverable, followed by identifying optimal screening methods for COPD in primary care. The greatest impact on COPD burden was associated with the idea of identifying strategies that are effective and cost-effective in reducing anxiety and depression among individuals with COPD. Finally, most of research ideas in the top 10 when considered by their likelihood of improving equity in the population were focused on low resource settings (**Table 3**). Please refer to the **Online Supplementary Document** for further details of top-ranked priorities and scores across each of the criteria.

**Table 3:** Top 3 research ideas by research criteria

Rank	Research idea	Answerable?	Effective?	Feasible?	Deliverable?	Impact?	Equity?	Rps	Aea
**ANSWERABILITY**									
1	Studying whether inhaled corticosteroids increase risk of bacterial infections in COPD	**1.000**	0.661	0.984	0.797	0.547	0.469	0.743	0.696
2	Defining criteria for antibiotics use in acute COPD	**0.970**	0.710	0.938	0.906	0.656	0.594	0.796	0.750
3	Agreeing on COPD definition that should be used for research aiming to have an impact on clinical practice globally	**0.970**	0.424	0.879	0.667	0.242	0.712	0.649	0.735
**EFFECTIVENESS**									
1	Developing new strategies (including new combinations of pharmacological and non-pharmacological strategies) to improve smoking cessation	0.952	**0.967**	0.790	0.790	0.903	0.806	0.868	0.779
2	Identifying optimal ways to detect smokers at risk of developing COPD and why only some of them seem to be at risk	0.803	**0.922**	0.625	0.550	0.703	0.594	0.699	0.627
3	Improved understanding of COPD risk factors and their association with COPD incidence and exact effects	0.894	**0.859**	0.984	0.797	0.734	0.734	0.834	0.770
**FEASIBILITY**									
1	Improved understanding of COPD risk factors and their association with COPD incidence and exact effects	0.894	0.859	**0.984**	0.797	0.734	0.734	0.834	0.770
2	Studying whether inhaled corticosteroids increase risk of bacterial infections in COPD	1.000	0.661	**0.984**	0.797	0.547	0.469	0.743	0.696
3	Identifying optimal screening method for COPD in primary care	0.938	0.773	**0.969**	0.924	0.667	0.848	0.853	0.814
**DELIVERABILITY**									
1	Identifying optimal diagnostic approach for COPD in low-resource settings	0.938	0.742	0.813	**0.938**	0.703	0.875	0.835	0.779
2	Identifying optimal screening method for COPD in primary care	0.938	0.773	0.969	**0.924**	0.667	0.848	0.853	0.814
3	Conducting trials to explore if early palliative care improves health outcomes in people with advanced COPD	0.919	0.650	0.968	**0.917**	0.400	0.633	0.748	0.691
**BURDEN**									
1	Identifying strategies that are effective and cost-effective in reducing anxiety and depression among individuals with COPD	0.933	0.583	0.919	0.800	**0.919**	0.742	0.816	0.672
2	Developing new strategies (including new combinations of pharmacological and non-pharmacological strategies) to improve smoking cessation	0.952	0.967	0.790	0.790	**0.903**	0.806	0.868	0.779
3	Identifying feasible and effective modes of delivery of pulmonary rehabilitation in low-resource settings	0.935	0.667	0.935	0.903	**0.823**	0.903	0.861	0.779
**EQUITABILITY**									
1	Defining the most affordable, accurate and reliable diagnostic process for respiratory symptoms in low-resource settings based on evidence	0.891	0.758	0.823	0.917	0.742	**0.935**	0.844	0.755
2	Identifying feasible and effective modes of delivery of pulmonary rehabilitation in low-resource settings	0.935	0.667	0.935	0.903	0.823	**0.903**	0.861	0.779
3	Identifying approached to scale-up of delivery of effective pulmonary rehabilitation in low resource settings to meet the burden of breathlessness	0.900	0.672	0.933	0.850	0.817	**0.900**	0.845	0.730

COPD – chronic obstructive pulmonary disease

## DISCUSSION

### Main findings

On the basis of the six suggested criteria, the top priority was focused on finding acceptable, effective and cost-effective ways for smoking cessation, which is research in risk reduction. Furthermore, three of the top 10 overall priorities called for research on improved screening and accurate diagnostic methods for COPD in primary care and low resource settings [42]. Moreover, six of the 20 top priorities were focused on feasible and cost-effective pulmonary rehabilitation delivery and access, particularly in primary care and low resource settings, which highlights the relevance of this exercise for LMICs. Other ideas that drew support from participants involved better understanding of risk factors for COPD, development of effective training programmes and guidelines for health workers and physicians in low resource settings and encouraging physical activity. The main output of the CHNRI process is an intuitive list of meaningful research questions provided by a group of 34 experts, with 230 research ideas organised and ranked according to explicit priority criteria. The COPD experts who took part in this CHNRI exercise concurred that most of these key research questions may be successfully answered by 2030.

### Findings in the context of the literature

A key difference between this process and those used in other prioritisation exercises, eg, the American Thoracic Society/European Respiratory Society statement on research questions in COPD [32] is that, in previous attempts to define research priorities, they were not systematically compared. Recommendations for research were usually listed as a result of broad overall consensus of participants following the discussion. Furthermore, the International Primary Care Respiratory Group (IPCRG) conducted an e-Delphi exercise, where diagnosing COPD in a primary care setting was a priority after that survey was completed. A notable difference was that community-based pulmonary rehabilitation was lower down the list and there was a separate category for tobacco dependence [33].

Meanwhile, in a UK priority setting for respiratory research involving physicians, researchers and professional societies [43], the leading focus leaned towards basic science including lung development and ageing, lung injury, repair and regeneration, susceptibility to infections, and sleep apnoea syndromes, which are not in our top priorities for reasons already discussed above. In an exercise involving only patients with COPD and asthma, priorities highlighted focused primarily on aetiology, co-morbidity and effective medication [44]. Another priority study among patients mainly emphasized the need to improve the ability to exercise among adults living with COPD [45]. Although both patients’ studies [44,45] employed no systematic measure of collating, analysing and ranking ideas, their findings reflect in our overall top priorities, and specifically for priorities listed under deliverability which largely focuses on pulmonary rehabilitation and physical activity, and addressing screening, diagnosis and treatment in primary care settings to improve outcomes, respectively.

### Research and policy implications

The experts agreed that the most pressing feasible research questions to address in the next decade for COPD reduction were on prevention, diagnosis and rehabilitation of COPD, especially in low resource settings. This appears to be in line with a number of recommendations from respiratory experts, giving credence to this exercise. For example, in a recent experts’ statement on the top ten research questions for improving COPD care in the next decade [46], they proposed exploring impact of new forms of electronic cigarettes on COPD burden, addressing challenges with COPD diagnosis, patient’s classification and risk stratification, and improvements in non-pharmacologic management particularly pulmonary rehabilitation, as top priorities, which clearly reflect in the our findings and support our recommendations for COPD research in the next decade. Important overall messages from the top of the list imply that LMIC settings should increasingly become a priority for research on COPD, because large majority of COPD-related deaths in the world today occur there. This implies an increased focus on low-cost preventive diagnostic and therapeutic measures, including tobacco reduction and increase in physical exercise.

Therefore, research on proper, cost-effective implementation of those measures will become increasingly important, which will require extensive capacity building in low-resource settings. Such research should be expected to lead to tailored country guidelines on addressing COPD, which are much needed [47]. Finally, the role of stress is still rather uncertain, but certainly worth investigating in future studies (**Box 3**) [48-50].

Box 3 **Possible role of stress**

Box topic not identified by the CHNRI method, which could be important to investigate for several reasons, is stress. Stress is a well-known trigger of inflammatory activity [48,49], and affects clinical outcomes in COPD [50], meaning that it is clinically relevant in this context, thus making stress a possible treatment target that could have benefits for COPD and beyond. Meanwhile, atmospheric pollution was broadly ranked as a non-smoking priority in the overall top 10 research priorities and also listed in the top ten by likelihood of effectively reducing the burden of COPD over time. However, it may be somewhat surprising that other specific areas of research on air quality particularly in LMICs, were not given more priority. After cigarette smoking, using biomass for cooking and the resulting indoor pollution is a major cause of COPD in less developed countries, where both indoor and outdoor air pollution are a concern worth studying further. Also, work on the development of pharmacological treatment or treating exacerbations did not feature prominently. Finally, although there were no specific questions about COVID-19 because the protocol for this exercise was developed in September 2019, the findings would still be relevant, and helpful at a follow-up to this exercise, in view of a likely risk for poor health outcomes among COVID-19 patients with COPD and other chronic airways diseases.

### Strengths and limitations

Diverse experts from across the world have come together for the first time to identify research priorities, which now need to be built on through strategic research investment in these prioritised areas. Nevertheless, some limitations are worth noting. The application of the CHNRI methodology to the field of COPD required some contextual adaptations including the experts and the scorers' selection, question consolidation, modified criteria and scoring processes. Still, it is important to note that the CHNRI methodology was originally conceived to be adaptive and has been similarly customised before and used in various settings [23], and our adaptations were collectively discussed and agreed upon by the Management Group. Another deviation from the prescribed process was lack of involvement from the representatives of the funders from the early stages.

In terms of equity, diversity and inclusivity, we carefully studied if the process inherently biased the outcomes against some groups or types of research questions. **Table 2** presented research questions that achieved the lowest scores. It is clear that highly theoretical work was not favoured in light of the urgent need to address the COPD burden globally, especially in LMICs. Besides, the criteria were chosen to prefer research questions that are feasible and could realistically address the burden within a decade. This did not favour research questions from basic science. This is why the questions at the bottom of the list were often highly specific, or advanced research ideas with more “blue-sky” thinking, or they had complex downstream outcomes.

One possible reason that could have disadvantaged highly specific or innovative research questions could be that some experts were not necessarily familiar enough with the research area to recognise a potentially feasible research question that is suitable for further exploration. It is also possible that professional expertise and clinical background of the experts affected the results. Furthermore, only COPD researchers were included, while other physicians (including those in primary care), nurses, patients, support groups and funding bodies, who are all important stakeholders, were not.

Despite efforts to represent all geographic regions and resource levels, respondents were predominantly COPD researchers from developed countries. We note that the South East Asia, African and Eastern Mediterranean regions were relatively under-represented. Although this likely reflects the current low levels of COPD research and advocacy efforts and potential barriers to participation that may exist in these settings, we acknowledge that priorities may vary by culture, region and resource level. This exercise was not able to capture and examine these differences, and culture and system specific research may be required.

Another important contributing factor for under-representation of LMIC-based researchers is likely the inclusion criterion of very high productivity or citations of their papers, which is difficult for them to achieve given limited resources for research, although our initial searches would have returned a sizeable number of researchers in HICs who were collaborating with colleagues in LMICs. This indeed reflects in our final top 20 rankings, with six focused on feasible and cost-effective pulmonary rehabilitation delivery and access in low resource settings, and three of the top 10 calling for research on improved screening and accurate diagnostic methods for COPD in primary care and low resource settings, both clearly important needs in LMICs. Moreover, seven of the research ideas in the top 10 under the likelihood of improving equity in the population were directly focused on LMICs (see Table S6 in the **Online Supplementary Document**). The drop-out rate from providing research questions to scoring them was also expected to be quite high. This is common with prioritisation exercises based on crowdsourcing, and it should be acknowledged, which was elaborated in **Box 1** [23-25].

## CONCLUSIONS

Research priorities identified by this systematic international process should inform and motivate policymakers, funders and researchers to support and conduct research to reduce the global burden of COPD. The largest gains should be expected in LMIC settings, as the large majority of COPD deaths occur in those settings [51]. The follow-up to this process should identify and map outcomes to currently funded research to highlight apparent gaps and opportunities for increased investment.

## Additional material


Online Supplementary Document
