# Supplementary material for: Research priorities to address the global burden of chronic obstructive pulmonary disease (COPD) in the next decade
Source: J Glob Health. 2021 Oct 9;11:15003. doi: 10.7189/jogh.11.15003 (PMC8542376; doi:10.7189/jogh.11.15003)

ONLINE SUPPLEMENTARY DOCUMENT

Research priorities to address the global burden of chronic obstructive pulmonary disease (COPD) in the next decade

TABLE OF CONTENTS

TABLE OF CONTENTS **2**

SUPPLEMENTARY TABLES **5**

Table S1: Top 10 research ideas by their likelihood of answerability. 5

Table S2: Top 10 research ideas by their likelihood of effectiveness. 6

Table S3: Top 10 research ideas by their likelihood of feasibility. 7

Table S4: Top 10 research ideas by their likelihood of deliverability. 8

Table S5: Top 10 research ideas by their likelihood of impact on COPD burden. 9

Table S6: Top 10 research ideas by their likelihood of equitability. 10

Table S7: Complete list of research ideas ranked by their overall Research Priority Scores (RPS) and corresponding Average Expert Agreement (AEA). 11

Table S8: Contributors to the Global COPD CHNRI exercise. 32

SUPPLEMENTARY FIGURES **34**

Figure S1. Flow chart of the COPD CHNRI research prioritization exercise 34

Top-ranked priorities across research criteria

Table 3 in the main paper presents the top 3 research ideas across each of the criteria. When research ideas were considered by their likelihood of answerability, it was notable that the proposed research idea "Studying whether inhaled corticosteroids increase risk of bacterial infections in COPD" received the maximum score. Also, the proposed research ideas to define criteria for antibiotics use in acute exacerbations of COPD, agree on COPD definition that should be used for research aiming to have an impact on clinical practice globally, and develop guidelines for health practitioners in low resource settings to diagnose and treat/manage their COPD patients were all seen as highly answerable. Interestingly, developing eHealth platforms to monitor adherence to and effectiveness of COPD medications in the community and conducting a trial of spirometry in routine primary care practice to screen for both early and adult lung function deficit were also seen as reasonably easy to answer.

For likelihood of effectiveness, there was very high agreement that developing new strategies (including new combinations of pharmacological and non-pharmacological strategies) to improve smoking cessation would be the most effective research idea. Several ideas linked with smoking were at the top, including the question on identifying optimal ways to detect smokers at risk of developing COPD and why only some of them seem to be at risk; improved understanding of COPD risk factors and their association with COPD incidence and exact effects; identifying optimal educational strategies for teenagers with nicotine addiction through smoking and vaping; exploring the non-smoking risk factors for the development of COPD; exploring the role of second-hand smoke, e-cigarettes and vaping as risk factors for COPD; and, identifying effective approaches to reduce exposure to passive smoking and indoor air pollution for children in low resource settings and assessing their impact. Clearly, there was a high level of consensus between experts that the impact of the proposed research on smoking would be the most effective way to address the global burden of COPD.

Among research ideas by their likelihood of feasibility, the leading research ideas were improved understanding of COPD risk factors and their association with COPD incidence and exact effects, studying whether inhaled corticosteroids increase the risk of bacterial infections in COPD, and identifying optimal screening methods for COPD in primary care. Ideas of health policy and systems research on feasibility of establishing pulmonary rehabilitation centres in the communities and improving the definition of COPD exacerbation based on an evidence-based protocol and its pathogenic mechanisms were also seen as very feasible. Two trials were also seen as very feasible: exploring if early palliative care improves health outcomes in people with advanced COPD and optimizing individualised use of inhaled corticosteroids in COPD to improve disease management and prevent side-effects. The idea of identifying feasible strategies to improve access to pulmonary rehabilitation had a very high average expert agreement.

When likelihood of deliverability was analysed, the experts viewed the proposed research idea of identifying optimal diagnostic approaches for COPD in low-resource settings as most deliverable,

followed by identifying optimal screening methods for COPD in primary care. This list also contains the research idea of identifying feasible and effective modes of delivery of pulmonary rehabilitation in low-resource settings.

In the collective opinion of our experts, the greatest impact on COPD burden could be achieved through identifying strategies that are effective and cost-effective in reducing anxiety and depression among individuals with COPD. This was followed by developing new strategies to improve smoking cessation and identifying feasible and effective modes of delivery of pulmonary rehabilitation in low-resource settings. An interesting idea that received a very high score was exploring whether "early" interventions (pharmacological and non-pharmacological) can stop or slow down the progression of COPD, followed by optimising treatment strategies for COPD patients with multi-morbidity.

Finally, most of research ideas in the top 10 when considered by their likelihood of improving equity in the population were focused on low resource settings. Examples include defining the most affordable, accurate and reliable evidence-based diagnostic process for respiratory symptoms in low-resource settings; and identifying feasible and effective modes of delivery of pulmonary rehabilitation in low-resource settings. These were followed by identifying approaches to scale-up of delivery of effective pulmonary rehabilitation in low resource settings to meet the burden of breathlessness and identifying the most cost-effective COPD treatment strategies for low resource settings. Please refer to the reminder of the **Appendix** for details of top-ranked priorities and scores across each of the criteria.

SUPPLEMENTARY TABLES

Table S1: Top 10 research ideas by their likelihood of answerability.

RANK	RESEARCH QUESTION	ANSWERABLE?	EFFECTIVE?	FEASIBLE?	DELIVERABLE?	IMPACT?	EQUITY?	RPS	AEA
1	Studying whether inhaled corticosteroids increase risk of bacterial infections in COPD	1.000	0.661	0.984	0.797	0.547	0.469	0.743	0.696
2	Defining criteria for antibiotics use in acute COPD	0.970	0.710	0.938	0.906	0.656	0.594	0.796	0.750
3	Agreeing on COPD definition that should be used for research aiming to have an impact on clinical practice globally	0.970	0.424	0.879	0.667	0.242	0.712	0.649	0.735
4	Developing guidelines for health practitioners in low resource settings to diagnose and treat/manage their COPD patients	0.969	0.700	0.875	0.906	0.633	0.766	0.808	0.745
5	Conducting a trial of spirometry in routine primary care practice as a screening measure of both early and adult lung function deficit	0.969	0.688	0.903	0.828	0.469	0.750	0.768	0.725
6	Developing eHealth platforms to monitor adherence to and effectiveness of COPD medications in the community	0.969	0.629	0.844	0.774	0.500	0.578	0.716	0.657
7	Determining the current level of knowledge and practices related to treatment of COPD among medical practitioners, in relation to national evidence-based guidelines	0.969	0.567	0.891	0.672	0.400	0.677	0.696	0.667
8	Studying if combining pharmacotherapy with exercise training improves cardiorespiratory function and reduces hospitalizations in patients with co-existing COPD and congestive heart failure	0.968	0.717	0.919	0.855	0.597	0.600	0.776	0.686
9	Identifying criteria to distinguish responders from non-responders for pulmonary rehabilitation in COPD?	0.968	0.467	0.903	0.871	0.569	0.548	0.721	0.657
10	Development of interventional strategies to improve or maintain physical activity	0.967	0.583	0.871	0.742	0.683	0.839	0.781	0.696

Table S2: Top 10 research ideas by their likelihood of effectiveness.

RANK	RESEARCH QUESTION	ANSWERABLE?	EFFECTIVE?	FEASIBLE?	DELIVERABLE?	IMPACT?	EQUITY?	RPS	AEA
1	Developing new strategies (including new combinations of pharmacological and non-pharmacological strategies) to improve smoking cessation	0.952	0.967	0.790	0.790	0.903	0.806	0.868	0.779
2	Identifying optimal ways to detect smokers at risk of developing COPD and why only some of them seem to be at risk	0.803	0.922	0.625	0.550	0.703	0.594	0.699	0.627
3	Improved understanding of COPD risk factors and their association with COPD incidence and exact effects	0.894	0.859	0.984	0.797	0.734	0.734	0.834	0.770
4	Identifying optimal educational strategies for teenagers on nicotine addiction through smoking and vaping	0.815	0.857	0.828	0.776	0.690	0.603	0.761	0.623
5	Exploring the non-smoking risk factors for the development of COPD (e.g., premature birth, childhood asthma, genes, biomass fumes exposure, atmospheric pollution, etc.)	0.909	0.855	0.844	0.797	0.774	0.875	0.842	0.775
6	Exploring the role of second-hand smoke, e-cigarettes and vaping as risk factors for COPD	0.875	0.823	0.903	0.633	0.629	0.629	0.749	0.676
7	Identifying effective approaches to reduce exposure to passive smoking and indoor air pollution for children in low resource settings and assessing their impact	0.800	0.817	0.726	0.758	0.567	0.774	0.740	0.647
8	Conducting long-term longitudinal trials of various preventing medications and lifestyle modifications in individuals at risk for COPD	0.742	0.806	0.597	0.548	0.710	0.700	0.684	0.608
9	Identifying optimal approaches to training physicians, healthcare workers, policy makers and the community in low resource settings about COPD and its risk factors	0.894	0.797	0.906	0.813	0.750	0.813	0.829	0.779
10	Investigating biological effects of high levels of ambient pollution (both indoors and outdoors) on COPD risk and progression	0.781	0.790	0.790	0.597	0.597	0.742	0.716	0.632

Table S3: Top 10 research ideas by their likelihood of feasibility.

RANK	RESEARCH QUESTION	ANSWERABLE?	EFFECTIVE?	FEASIBLE?	DELIVERABLE?	IMPACT?	EQUITY?	RPS	AEA
1	Improved understanding of COPD risk factors and their association with COPD incidence and exact effects	0.894	0.859	0.984	0.797	0.734	0.734	0.834	0.770
2	Studying whether inhaled corticosteroids increase risk of bacterial infections in COPD	1.000	0.661	0.984	0.797	0.547	0.469	0.743	0.696
3	Identifying optimal screening method for COPD in primary care	0.938	0.773	0.969	0.924	0.667	0.848	0.853	0.814
4	Health policy and systems research on feasibility of establishing pulmonary rehabilitation centers in the communities	0.906	0.645	0.969	0.891	0.742	0.781	0.822	0.765
5	Improving the definition of COPD exacerbation based on an evidence-based protocol and its pathogenic mechanisms	0.924	0.594	0.969	0.742	0.375	0.606	0.702	0.706
6	Conducting trials to explore if early palliative care improves health outcomes in people with advanced COPD	0.919	0.650	0.968	0.917	0.400	0.633	0.748	0.691
7	Conducting trials to optimize individualized use of inhaled corticosteroids in COPD to improve disease management and prevent side effects	0.935	0.683	0.966	0.793	0.433	0.583	0.732	0.647
8	Identifying feasible strategies to improve access to pulmonary rehabilitation for COPD patients whilst retaining cost effectiveness	0.953	0.710	0.953	0.891	0.766	0.813	0.847	0.779
9	Studying the effectiveness of physical activity incentive programs on prevention of hospitalizations due to acute exacerbations of COPD	0.903	0.694	0.952	0.774	0.625	0.742	0.782	0.711
10	Defining criteria for antibiotics use in acute COPD	0.970	0.710	0.938	0.906	0.656	0.594	0.796	0.750

Table S4: Top 10 research ideas by their likelihood of deliverability.

RANK	RESEARCH QUESTION	ANSWERABLE?	EFFECTIVE?	FEASIBLE?	DELIVERABLE?	IMPACT?	EQUITY?	RPS	AEA
1	Identifying optimal diagnostic approach for COPD in low-resource settings	0.938	0.742	0.813	0.938	0.703	0.875	0.835	0.779
2	Identifying optimal screening method for COPD in primary care	0.938	0.773	0.969	0.924	0.667	0.848	0.853	0.814
3	Conducting trials to explore if early palliative care improves health outcomes in people with advanced COPD	0.919	0.650	0.968	0.917	0.400	0.633	0.748	0.691
4	Defining the most affordable, accurate and reliable diagnostic process for respiratory symptoms in low-resource settings based on evidence	0.891	0.758	0.823	0.917	0.742	0.935	0.844	0.755
5	Defining criteria for antibiotics use in acute COPD	0.970	0.710	0.938	0.906	0.656	0.594	0.796	0.750
6	Developing guidelines for health practitioners in low resource settings to diagnose and treat/manage their COPD patients	0.969	0.700	0.875	0.906	0.633	0.766	0.808	0.745
7	Identifying feasible and effective modes of delivery of pulmonary rehabilitation in low-resource settings	0.935	0.667	0.935	0.903	0.823	0.903	0.861	0.779
8	Identifying who, and when, should be treated with inhaled corticosteroids in COPD	0.933	0.586	0.833	0.900	0.567	0.700	0.753	0.652
9	Health policy and systems research on feasibility of establishing pulmonary rehabilitation centers in the communities	0.906	0.645	0.969	0.891	0.742	0.781	0.822	0.765
10	Identifying feasible strategies to improve access to pulmonary rehabilitation for COPD patients whilst retaining cost effectiveness	0.953	0.710	0.953	0.891	0.766	0.813	0.847	0.779

Table S5: Top 10 research ideas by their likelihood of impact on COPD burden.

RANK	RESEARCH QUESTION	ANSWERABLE?	EFFECTIVE?	FEASIBLE?	DELIVERABLE?	IMPACT?	EQUITY?	RPS	AEA
1	Identifying strategies that are effective and cost-effective in reducing anxiety and depression among individuals with COPD	0.933	0.583	0.919	0.800	0.919	0.742	0.816	0.672
2	Developing new strategies (including new combinations of pharmacological and non-pharmacological strategies) to improve smoking cessation	0.952	0.967	0.790	0.790	0.903	0.806	0.868	0.779
3	Identifying feasible and effective modes of delivery of pulmonary rehabilitation in low-resource settings	0.935	0.667	0.935	0.903	0.823	0.903	0.861	0.779
4	Identifying approached to scale-up of delivery of effective pulmonary rehabilitation in low resource settings to meet the burden of breathlessness	0.900	0.672	0.933	0.850	0.817	0.900	0.845	0.730
5	Exploring whether "early" interventions (pharmacological and non-pharmacological) can stop or slow down the progression of COPD	0.839	0.767	0.767	0.677	0.793	0.700	0.757	0.672
6	Optimizing treatment strategies for multimorbid COPD	0.823	0.645	0.766	0.726	0.781	0.767	0.751	0.652
7	Exploring the non-smoking risk factors for the development of COPD (e.g., premature birth, childhood asthma, genes, biomass fumes exposure, atmospheric pollution, etc.)	0.909	0.855	0.844	0.797	0.774	0.875	0.842	0.775
8	Identifying feasible strategies to improve access to pulmonary rehabilitation for COPD patients whilst retaining cost effectiveness	0.953	0.710	0.953	0.891	0.766	0.813	0.847	0.779
9	Identifying optimal approaches to training physicians, healthcare workers, policy makers and the community in low resource settings about COPD and its risk factors	0.894	0.797	0.906	0.813	0.750	0.813	0.829	0.779
10	Defining the most affordable, accurate and reliable diagnostic process for respiratory symptoms in low-resource settings based on evidence	0.891	0.758	0.823	0.917	0.742	0.935	0.844	0.755

Table S6: Top 10 research ideas by their likelihood of equitability.

RANK	RESEARCH QUESTION	ANSWERABLE?	EFFECTIVE?	FEASIBLE?	DELIVERABLE?	IMPACT?	EQUITY?	RPS	AEA
1	Defining the most affordable, accurate and reliable diagnostic process for respiratory symptoms in low-resource settings based on evidence	0.891	0.758	0.823	0.917	0.742	0.935	0.844	0.755
2	Identifying feasible and effective modes of delivery of pulmonary rehabilitation in low-resource settings	0.935	0.667	0.935	0.903	0.823	0.903	0.861	0.779
3	Identifying approached to scale-up of delivery of effective pulmonary rehabilitation in low resource settings to meet the burden of breathlessness	0.900	0.672	0.933	0.850	0.817	0.900	0.845	0.730
4	Exploring the non-smoking risk factors for the development of COPD (e.g., premature birth, childhood asthma, genes, biomass fumes exposure, atmospheric pollution, etc.)	0.909	0.855	0.844	0.797	0.774	0.875	0.842	0.775
5	Identifying optimal diagnostic approach for COPD in low-resource settings	0.938	0.742	0.813	0.938	0.703	0.875	0.835	0.779
6	Exploring if COPD treatment in low- and middle-income countries be substantially different because of large differences in risk profile	0.742	0.578	0.781	0.703	0.609	0.875	0.715	0.652
7	Identifying the most cost-effective COPD treatment strategies for low resource settings	0.903	0.700	0.806	0.806	0.733	0.871	0.803	0.725
8	Encouraging prioritization of COPD within the public health system in low-resource settings	0.571	0.724	0.707	0.667	0.633	0.871	0.696	0.583
9	Identifying optimal screening method for COPD in primary care	0.938	0.773	0.969	0.924	0.667	0.848	0.853	0.814
10	Identifying difficulties to diagnose and manage COPD for general practitioners in a rural area	0.906	0.656	0.828	0.813	0.500	0.844	0.758	0.706

Table S7: Complete list of research ideas ranked by their overall Research Priority Scores (RPS) and corresponding Average Expert Agreement (AEA).

RANK	RESEARCH IDEA	SUB-THEME	ANSWER ABLE?	EFFECTI VE?	FEASIBL E?	DELIVER ABLE?	IMPACT ?	EQUITY?	RPS	AEA
1	Developing new strategies (including new combinations of pharmacological and non-pharmacological strategies) to improve smoking cessation	III	0.952	0.967	0.790	0.790	0.903	0.806	0.868	0.779
2	Identifying feasible and effective modes of delivery of pulmonary rehabilitation in low-resource settings	III	0.935	0.667	0.935	0.903	0.823	0.903	0.861	0.779
3	Identifying optimal screening method for COPD in primary care	V	0.938	0.773	0.969	0.924	0.667	0.848	0.853	0.814
4	Identifying feasible strategies to improve access to pulmonary rehabilitation for COPD patients whilst retaining cost effectiveness	III	0.953	0.710	0.953	0.891	0.766	0.813	0.847	0.779
5	Identifying approached to scale-up of delivery of effective pulmonary rehabilitation in low resource settings to meet the burden of breathlessness	III	0.900	0.672	0.933	0.850	0.817	0.900	0.845	0.730
6	Defining the most affordable, accurate and reliable diagnostic process for respiratory symptoms in low-resource settings based on evidence	V	0.891	0.758	0.823	0.917	0.742	0.935	0.844	0.755
7	Exploring the non-smoking risk factors for the development of COPD (e.g., premature birth, childhood asthma, genes, biomass fumes exposure, atmospheric pollution, etc.)	I	0.909	0.855	0.844	0.797	0.774	0.875	0.842	0.775
8	Identifying optimal diagnostic approach for COPD in low-resource settings	V	0.938	0.742	0.813	0.938	0.703	0.875	0.835	0.779
9	Improved understanding of COPD risk factors and their association with COPD incidence and exact effects	I	0.894	0.859	0.984	0.797	0.734	0.734	0.834	0.770

10	Identifying optimal approaches to training physicians, healthcare workers, policy makers and the community in low resource settings about COPD and its risk factors	III	0.894	0.797	0.906	0.813	0.750	0.813	0.829	0.779
11	Health policy and systems research on feasibility of establishing pulmonary rehabilitation centers in the communities	III	0.906	0.645	0.969	0.891	0.742	0.781	0.822	0.765
12	Identifying strategies that are effective and cost-effective in reducing anxiety and depression among individuals with COPD	III	0.933	0.583	0.919	0.800	0.919	0.742	0.816	0.672
13	Developing guidelines for health practitioners in low resource settings to diagnose and treat/manage their COPD patients	V	0.969	0.700	0.875	0.906	0.633	0.766	0.808	0.745
14	Identifying the most cost-effective COPD treatment strategies for low resource settings	III	0.903	0.700	0.806	0.806	0.733	0.871	0.803	0.725
15	Defining criteria for antibiotics use in acute COPD	III	0.970	0.710	0.938	0.906	0.656	0.594	0.796	0.750
16	Developing an eHealth pulmonary rehabilitation program that is as effective as regular rehabilitation in both health benefits and costs	III	0.938	0.661	0.906	0.875	0.703	0.688	0.795	0.740
17	Identifying the most effective e-health, m-health and telemedicine interventions for the management of COPD in primary care	III	0.938	0.613	0.906	0.891	0.719	0.688	0.792	0.735
18	Identifying feasible and effective means to increase physical activity in patients with newly diagnosed COPD	III	0.919	0.661	0.891	0.839	0.629	0.806	0.791	0.711
19	Identifying feasible approaches for providing pulmonary rehabilitation in primary care	III	0.935	0.567	0.903	0.823	0.710	0.774	0.785	0.711
20	Studying the effectiveness of physical activity incentive programs on prevention of hospitalizations due to acute exacerbations of COPD	III	0.903	0.694	0.952	0.774	0.625	0.742	0.782	0.711
21	Development of interventional strategies to improve or maintain physical activity	III	0.967	0.583	0.871	0.742	0.683	0.839	0.781	0.696

22	Conducting trials to compare standard strategies of pharmacological treatment and combinations of pharmacological and non-pharmacological (rehabilitation) strategies to slow the rate of decline in lung function	VI	0.903	0.733	0.933	0.800	0.650	0.655	0.779	0.681
23	Studying the effectiveness of pulmonary rehabilitation in individuals with COPD and multiple comorbidities	III	0.938	0.613	0.938	0.875	0.625	0.672	0.777	0.725
24	Identifying biomarkers to guide management of COPD exacerbations (systemic steroid vs. antibiotic treatment)	II	0.967	0.767	0.897	0.810	0.583	0.633	0.776	0.667
25	Studying if combining pharmacotherapy with exercise training improves cardiorespiratory function and reduces hospitalizations in patients with co-existing COPD and congestive heart failure	III	0.968	0.717	0.919	0.855	0.597	0.600	0.776	0.686
26	Conducting a cluster-randomized trial comparing alternative strategies to increase participation in pulmonary rehabilitation following hospital discharge	IV	0.909	0.742	0.922	0.875	0.531	0.645	0.771	0.721
27	Defining epidemiological characteristics of COPD in Africa in terms of possible effects of chronic infections, nutrition and environmental pollution	I	0.906	0.742	0.774	0.750	0.613	0.839	0.771	0.681
28	Conducting a trial of spirometry in routine primary care practice as a screening measure of both early and adult lung function deficit	V	0.969	0.688	0.903	0.828	0.469	0.750	0.768	0.725
29	Defining epidemiological characteristics of COPD in Africa in terms of possible under-reporting, effects of smoking and biomass, and urban-rural differences	I	0.922	0.694	0.790	0.717	0.629	0.839	0.765	0.686
30	Identifying optimal educational strategies for teenagers on nicotine addiction through smoking and vaping	III	0.815	0.857	0.828	0.776	0.690	0.603	0.761	0.623
31	Identifying difficulties to diagnose and manage COPD for general practitioners in a rural area	V	0.906	0.656	0.828	0.813	0.500	0.844	0.758	0.706

32	Exploring whether "early" interventions (pharmacological and non-pharmacological) can stop or slow down the progression of COPD	VI	0.839	0.767	0.767	0.677	0.793	0.700	0.757	0.672
33	Identifying who, and when, should be treated with inhaled corticosteroids in COPD	III	0.933	0.586	0.833	0.900	0.567	0.700	0.753	0.652
34	Optimizing treatment strategies for multimorbid COPD	III	0.823	0.645	0.766	0.726	0.781	0.767	0.751	0.652
35	Assessing the impact of concomitant diseases on the effectiveness of COPD treatment	III	0.919	0.600	0.887	0.726	0.629	0.742	0.751	0.662
36	Exploring the role of second-hand smoke, e-cigarettes and vaping as risk factors for COPD	I	0.875	0.823	0.903	0.633	0.629	0.629	0.749	0.676
37	Conducting trials to explore if early palliative care improves health outcomes in people with advanced COPD	III	0.919	0.650	0.968	0.917	0.400	0.633	0.748	0.691
38	Identifying the barriers and facilitators to wider uptake and completion of pulmonary rehabilitation	III	0.844	0.645	0.844	0.742	0.625	0.781	0.747	0.696
39	Studying whether inhaled corticosteroids increase risk of bacterial infections in COPD	III	1.000	0.661	0.984	0.797	0.547	0.469	0.743	0.696
40	Identifying effective approaches to reduce exposure to passive smoking and indoor air pollution for children in low resource settings and assessing their impact	I	0.800	0.817	0.726	0.758	0.567	0.774	0.740	0.647
41	Identifying environmental factors that accelerate lung function decline in COPD	I	0.845	0.768	0.750	0.600	0.717	0.750	0.738	0.623
42	Exploring the feasibility of spirometry screening of general population at an early age	V	0.938	0.641	0.891	0.672	0.531	0.750	0.737	0.686
43	Studying if anti-oxidants reduce exacerbations frequency in COPD	VI	0.966	0.574	0.897	0.845	0.554	0.571	0.734	0.603
44	Conducting trials to optimize individualized use of inhaled corticosteroids in COPD to improve disease management and prevent side effects	V	0.935	0.683	0.966	0.793	0.433	0.583	0.732	0.647

45	Assessing the impact of successful treatment of comorbid diseases associated with COPD on the course of the lung disease (and vice versa)	VI	0.768	0.607	0.776	0.821	0.607	0.793	0.729	0.603
46	Investigating the effectiveness of teleconsultation in management of COPD cases once the case is diagnosed and started on treatment (i.e. maintenance phase)	III	0.952	0.661	0.935	0.758	0.532	0.532	0.728	0.652
47	Studying the effect of peer coaching / support intervention on rates of participation in pulmonary rehabilitation in COPD	IV	0.903	0.600	0.935	0.806	0.452	0.645	0.724	0.672
48	Exploring if impending exacerbations can be reliably predicted and prevented based on home-monitoring data	IV	0.931	0.638	0.759	0.817	0.600	0.583	0.721	0.618
49	Identifying criteria to distinguish responders from non-responders for pulmonary rehabilitation in COPD?	III	0.968	0.467	0.903	0.871	0.569	0.548	0.721	0.657
50	Developing and validating an individualized clinical prediction tool that combines patient (e.g., age, sex, smoking history, COPD history) and disease characteristics (e.g., exacerbation frequency, lung function, EOS count) to determine the most optimal pharmacotherapy for each patient	V	0.828	0.700	0.694	0.707	0.742	0.645	0.719	0.613
51	Identifying the most efficient strategies for screening or case detection of undiagnosed COPD and exploring the benefits of implementing such a strategy	V	0.790	0.606	0.766	0.733	0.594	0.813	0.717	0.657
52	Investigating biological effects of high levels of ambient pollution (both indoors and outdoors) on COPD risk and progression	I	0.781	0.790	0.790	0.597	0.597	0.742	0.716	0.632
53	Developing eHealth platforms to monitor adherence to and effectiveness of COPD medications in the community	IV	0.969	0.629	0.844	0.774	0.500	0.578	0.716	0.657
54	Exploring if COPD treatment in low- and middle-income countries be substantially different because of large differences in risk profile	I	0.742	0.578	0.781	0.703	0.609	0.875	0.715	0.652

55	Defining the physiological parameters for safe discharge from hospital following an acute exacerbation of COPD to prevent repeated admissions	V	0.844	0.563	0.891	0.844	0.484	0.656	0.714	0.672
56	Designing and establishing National COPD Control Programs that focus on public health systems and public engagement in COPD diagnosis, treatment and management	III	0.750	0.650	0.733	0.733	0.667	0.733	0.711	0.613
57	Comparing biomass-related COPD and cigarette-related COPD and optimizing their treatment accordingly	III	0.844	0.516	0.781	0.781	0.500	0.813	0.706	0.652
58	Identifying cost-effective strategies to enhance medication adherence and the optimal ways of their implementation in daily practice	IV	0.914	0.603	0.867	0.683	0.567	0.600	0.706	0.593
59	Identifying optimal modes of delivery of rehabilitation and integrated programme for COPD (e.g. the content, timing, duration, types of exercises, follow up time and outcome measures)	III	0.806	0.567	0.935	0.790	0.548	0.581	0.705	0.637
60	Improving the definition of COPD exacerbation based on an evidence-based protocol and its pathogenic mechanisms	II	0.924	0.594	0.969	0.742	0.375	0.606	0.702	0.706
61	Studying if macrolides without anti-bacterial activities boost anti-viral immunity and prevent exacerbations in COPD	III	0.833	0.569	0.867	0.767	0.603	0.567	0.701	0.593
62	Identifying the key components (duration, location and content) of pulmonary rehabilitation programmes that have the greatest impact on the lives of individuals with COPD	III	0.797	0.581	0.844	0.797	0.563	0.625	0.701	0.652
63	Identifying effective treatments for mucus hypersecretion	III	0.933	0.517	0.867	0.783	0.550	0.554	0.701	0.578
64	Identifying optimal ways to detect smokers at risk of developing COPD and why only some of them seem to be at risk	V	0.803	0.922	0.625	0.550	0.703	0.594	0.699	0.627
65	Developing novel medications to halt or prevent progression of COPD	III	0.810	0.759	0.482	0.741	0.732	0.667	0.699	0.534

66	Investigating a role for vaccines against bacteria and viruses that contribute to disease exacerbations in COPD patients	III	0.845	0.655	0.750	0.650	0.655	0.633	0.698	0.593
67	Determining the current level of knowledge and practices related to treatment of COPD among medical practitioners, in relation to national evidence-based guidelines	III	0.969	0.567	0.891	0.672	0.400	0.677	0.696	0.667
68	Encouraging prioritization of COPD within the public health system in low-resource settings	III	0.571	0.724	0.707	0.667	0.633	0.871	0.696	0.583
69	Identifying effective strategies to detect underlying anxiety and depression in people with COPD	III	0.900	0.550	0.839	0.733	0.435	0.710	0.695	0.637
70	Exploring whether integration of telemonitoring and computer-based decision support systems is effective for the improvement of self-management and prevention of exacerbations in COPD	IV	0.871	0.516	0.828	0.790	0.547	0.609	0.694	0.627
71	Exploring the effects of pharmacotherapy in patients with early-mild COPD on day-to-day patient care and the natural history of COPD	VI	0.867	0.661	0.732	0.724	0.589	0.586	0.693	0.578
72	Evaluating the effectiveness of empirical antimicrobial therapy in acute exacerbations of COPD	III	0.966	0.625	0.839	0.759	0.466	0.466	0.687	0.583
73	Improving the 'transferability' of pulmonary rehabilitation benefits towards daily life	III	0.724	0.556	0.724	0.778	0.571	0.759	0.685	0.559
74	Identifying the optimal ways of implementation of digital/electronic diagnostic devices and inhalers in COPD patients	V	0.867	0.621	0.817	0.733	0.567	0.500	0.684	0.583
75	Conducting long-term longitudinal trials of various preventing medications and lifestyle modifications in individuals at risk for COPD	III	0.742	0.806	0.597	0.548	0.710	0.700	0.684	0.608
76	Assessing the need for, and effectiveness of, patient-centred digital tools for monitoring symptoms and adherence to treatment	V	0.844	0.581	0.813	0.828	0.531	0.484	0.680	0.623

77	Identifying the causes of COPD exacerbations in different global regions and what are the key differences in exacerbation phenotypes	II	0.804	0.571	0.700	0.717	0.431	0.833	0.676	0.583
78	Exploring preventive strategies to avoid decline of lung function at an early age	III	0.768	0.603	0.650	0.589	0.655	0.786	0.675	0.549
79	Investigating if vaping alters the pathophysiology of COPD in comparison to cigarette smoking	II	0.875	0.613	0.935	0.726	0.435	0.452	0.673	0.642
80	Identifying mechanistic pathways underlying acute exacerbations of COPD and their effect on optimal management of exacerbations	III	0.897	0.603	0.817	0.650	0.600	0.433	0.667	0.583
81	Identifying optimal ways to detect early COPD before fixed airways obstruction occurs	V	0.758	0.774	0.581	0.650	0.583	0.645	0.665	0.588
82	Assessing the effectiveness of different medical treatments of COPD	III	0.862	0.643	0.793	0.655	0.414	0.621	0.665	0.588
83	Identifying optimal strategies for promoting healthy lung aging	III	0.643	0.722	0.603	0.569	0.732	0.690	0.660	0.534
84	Identifying modifiable and non-modifiable determinants of COPD	I	0.788	0.703	0.750	0.547	0.563	0.594	0.657	0.613
85	Conducting trials to explore of LABA/LAMA/IC therapy prevents severe exacerbations better than LAMA/LABA, or than LAMA or LABA alone	III	0.917	0.667	0.850	0.667	0.350	0.483	0.656	0.603
86	Identifying the optimal ways of contribution of ASHA (or other public health representatives) to the diagnosis and management of COPD at the community level?	V	0.776	0.574	0.786	0.679	0.463	0.638	0.653	0.534
87	Identifying delivery modes for supportive or palliative care within routine care in people with increasingly symptomatic COPD	III	0.845	0.500	0.783	0.700	0.417	0.667	0.652	0.578
88	Conducting large, prospective and multivariate epidemiological studies to understand the relative effect size of different categories of COPD - by age, environmental factors, maternal factors, gene-environmental factors and concomitant diseases	I	0.828	0.597	0.694	0.613	0.468	0.710	0.651	0.593

89	Agreeing on COPD definition that should be used for research aiming to have an impact on clinical practice globally	V	0.970	0.424	0.879	0.667	0.242	0.712	0.649	0.735
90	Evaluating the effectiveness and cost-effectiveness of digital/electronic diagnostic devices and inhalers in COPD patients	V	0.903	0.516	0.758	0.661	0.532	0.516	0.648	0.574
91	Exploring the overlap between COPD and coronary heart disease in terms of shared pathophysiology and treatment implications	III	0.919	0.467	0.900	0.741	0.300	0.550	0.646	0.618
92	Defining types of COPD exacerbations by severity and endotype-phenotype relationships	II	0.922	0.375	0.859	0.734	0.452	0.531	0.646	0.647
93	Conducting long-term epidemiological studies to understand the life course of COPD, based on adolescents and young adults at risk	I	0.727	0.750	0.625	0.468	0.656	0.645	0.645	0.593
94	Establishing the spirometry cut-off values for diagnosing COPD in different ethnic groups	V	0.859	0.387	0.859	0.734	0.258	0.750	0.641	0.696
95	Exploring the impact of systemic inflammation in COPD	II	0.914	0.500	0.717	0.655	0.448	0.600	0.639	0.544
96	Measuring the impact of 'childhood disadvantage factors' on COPD in later life	VI	0.707	0.625	0.683	0.554	0.429	0.828	0.637	0.544
97	Exploring the contribution of the lung microbiome to COPD and its progression	II	0.914	0.593	0.800	0.633	0.345	0.533	0.636	0.588
98	Exploring whether inhalation antibiotics prevent COPD exacerbations	III	0.817	0.617	0.800	0.667	0.350	0.567	0.636	0.598
99	Developing point-of-care tests to effectively separate different types of COPD exacerbations in a community	V	0.862	0.533	0.650	0.694	0.484	0.594	0.636	0.569
100	Investigating if therapeutic intervention with inhaled interferon (beta or lambda) ameliorates exacerbation severity in COPD	III	0.926	0.482	0.857	0.696	0.444	0.407	0.636	0.544
101	Exploring the effect of a peer coaching / support intervention on outcomes of COPD	III	0.931	0.466	0.800	0.683	0.383	0.550	0.636	0.588

102	Studying how treatment efficacy differs between COPD caused by different risk factors	I	0.742	0.581	0.734	0.629	0.469	0.656	0.635	0.593
103	Exploring if accelerated senescence has a major pathogenetic role in the progression of stable COPD	II	0.880	0.519	0.815	0.519	0.537	0.537	0.635	0.471
104	Developing biomarkers to predict exacerbations, especially severe ones	V	0.714	0.643	0.672	0.655	0.534	0.586	0.634	0.520
105	Studying how prognosis differs between COPD caused by different risk factors	VI	0.919	0.467	0.862	0.517	0.367	0.655	0.631	0.593
106	Exploring the impact of specific comorbidities on the quality of life and neurocognitive functions among COPD patients in low- and middle-income countries	III	0.862	0.370	0.867	0.567	0.333	0.783	0.630	0.608
107	Exploring the possible role of human microbiome in COPD patients	II	0.900	0.556	0.806	0.629	0.367	0.516	0.629	0.583
108	Identifying effective and cost-effective strategies in reducing the psychosocial burden of caring for a COPD patient	III	0.724	0.466	0.800	0.683	0.483	0.617	0.629	0.544
109	Identifying quantitative imaging features that can be used and standardised to assess COPD disease progression and treatment response	VI	0.903	0.483	0.823	0.726	0.317	0.516	0.628	0.613
110	Exploring how disease progression in COPD, assessed by spirometry and imaging, varies across different global regions and what are the key factors that influence variation	VI	0.800	0.517	0.726	0.645	0.433	0.645	0.628	0.574
111	Developing international registries or electronic health records to enable "big data analytics" as a tool to generate working hypothesis for future researches	V	0.855	0.532	0.672	0.672	0.531	0.500	0.627	0.569
112	Developing and evaluating a complex intervention (including pharamacological and non-pharmacological supporting strategies) for COPD patients to prevent / cope with episodic breathlessness	III	0.839	0.593	0.655	0.625	0.466	0.571	0.625	0.520

113	Identifying biomarkers for predicting response to ICS in patients with COPD	VI	0.786	0.534	0.690	0.655	0.433	0.621	0.620	0.544
114	Investigating the potential for small molecule anti-inflammatory treatment to halt disease progression and restore pulmonary homeostasis	III	0.839	0.643	0.732	0.519	0.448	0.533	0.619	0.515
115	Studying the impact of the polypill (hydrochlorothiazide 12·5 mg, aspirin 81 mg, atorvastatin 20 mg, and enalapril 5 mg or valsartan 40 mg) on mortality in COPD patients	III	0.911	0.464	0.804	0.661	0.426	0.448	0.619	0.539
116	Conducting a randomized controlled trial of available inhaled triple combinations consisting of inhaled corticosteroids, long-acting beta-agonists and long-acting muscarinic antagonists (ICS, LABA, LAMA) with the outcomes defined as reduction of exacerbations and safety from pneumonia risk	III	0.900	0.500	0.893	0.732	0.276	0.400	0.617	0.603
117	Social science studies to understand the psychological and physical/lifestyle responses of young adults labelled as at long-term risk of COPD development	I	0.600	0.656	0.844	0.550	0.435	0.613	0.616	0.574
118	Advancing precision medicine to identify disease endotypes that respond better to different treatments	V	0.879	0.617	0.733	0.621	0.345	0.500	0.616	0.574
119	Establishing new indications for non-invasive mechanical ventilation in COPD and oxygen	III	0.914	0.517	0.672	0.655	0.400	0.533	0.615	0.539
120	Investigating if prophylactic therapy with inhaled interferon (beta or lambda) prevents exacerbations in COPD	III	0.857	0.536	0.786	0.661	0.407	0.444	0.615	0.529
121	Studying the natural history of airflow obstruction and emphysema in young adults	II	0.852	0.518	0.810	0.611	0.375	0.517	0.614	0.525
122	Establishing reproducible blood and sputum-based biomarkers predictive of clinical progression of COPD	V	0.750	0.534	0.776	0.600	0.483	0.533	0.613	0.529
123	Defining the annual loss of lung function in high risk individuals according to established criteria of COPD risk	II	0.906	0.516	0.903	0.565	0.383	0.400	0.612	0.618

124	Identifying biomarkers useful as intermediate targets to guide drug development	V	0.778	0.607	0.661	0.536	0.554	0.536	0.612	0.490
125	Determining the proportion of patients with COPD who can demonstrate good knowledge and practices in self-care of COPD	IV	0.800	0.400	0.839	0.565	0.484	0.581	0.611	0.583
126	Conducting translational research to identify promising pharmacological agents for an effective relief of chronic breathlessness in patients with COPD	III	0.817	0.517	0.655	0.603	0.589	0.481	0.611	0.490
127	Studying the role of chronic bronchial infection by potentially pathogenic microorganisms in stable COPD	II	0.870	0.552	0.741	0.569	0.379	0.552	0.610	0.529
128	Defining the relationship between the inflammatory load, a stable and an exacerbated status	II	0.883	0.417	0.879	0.617	0.417	0.417	0.605	0.588
129	Exploring the effectiveness of High Sensitivity (HS) troponin and NT pro-BNP after first diagnosis of COPD to better stratify mortality risk and treat cardiovascular comorbidity?	V	0.917	0.483	0.857	0.633	0.400	0.333	0.604	0.593
130	Identifying mechanisms of secondary respiratory bacterial infections following viral infections in COPD	II	0.796	0.537	0.750	0.625	0.362	0.552	0.604	0.525
131	Exploring the implications and cause of airways bacterial colonisation	II	0.804	0.519	0.776	0.714	0.250	0.550	0.602	0.564
132	Exploring whether immune stimulants reduce exacerbations in COPD	II	0.821	0.464	0.793	0.518	0.536	0.444	0.596	0.500
133	Explaining the role of the eosinophilic inflammation and circulating eosinophils as a biomarker of exacerbations, other endpoints and response to treatment	V	0.850	0.429	0.733	0.724	0.350	0.483	0.595	0.549
134	Exploring a need for, and feasibility of, a national control program for COPD, based on perspectives and perceptions of stakeholders at various levels	III	0.661	0.483	0.667	0.633	0.483	0.629	0.593	0.520

135	Estimating the prevalence of airflow obstruction and/or respiratory symptoms (ie., early COPD) in middle-aged smokers and vapers and factors that affect disease progression in both groups 5 years later	I	0.817	0.431	0.783	0.717	0.290	0.516	0.592	0.593
136	Redefining GOLD treatment guidelines based on symptoms, exacerbations and responders/non-responders to steroids, while abandoning staging criteria	III	0.766	0.433	0.774	0.694	0.333	0.548	0.591	0.588
137	Studying the mechanisms of increased susceptibility to respiratory viral infections in COPD	II	0.788	0.500	0.722	0.607	0.393	0.536	0.591	0.495
138	Conducting trials to explore whether PDE4 inhibitors (eg, roflumilast) improve quality of life in patients with eosinophilic COPD in addition to triple therapy in GOLD patients	III	0.903	0.550	0.867	0.650	0.267	0.300	0.589	0.642
139	Studying how health professionals can work more closely with patients to understand their concerns and fears	III	0.645	0.484	0.688	0.613	0.419	0.688	0.589	0.549
140	Developing novel symptomatic treatments to relieve chronic cough and sputum production	III	0.845	0.534	0.661	0.638	0.293	0.552	0.587	0.544
141	Identifying clinical, physiological or biochemical markers of COPD that can be tele-monitored to predict acute exacerbations	VI	0.732	0.464	0.586	0.586	0.600	0.550	0.586	0.500
142	Studying the difference in etiology and biomarkers for non-smoking-related COPD (eg., genetic, biomass, infections, asthma, abnormal lung development, TB or HIV-related)	V	0.833	0.429	0.804	0.607	0.304	0.536	0.585	0.529
143	Exploring if novel non-antibiotic means of bacterial control could enhance innate immune activation in COPD	III	0.780	0.500	0.692	0.519	0.481	0.519	0.582	0.446
144	Developing new anti-inflammatory treatments	III	0.690	0.643	0.554	0.518	0.603	0.483	0.582	0.475
145	Separating disease activity from stable impairment to define risk of progression in COPD	I	0.677	0.700	0.726	0.586	0.435	0.355	0.580	0.554

146	Identifying effective health services delivery models for people with COPD approaching the end of life	III	0.690	0.433	0.677	0.567	0.484	0.613	0.577	0.520
147	Conducting pathophysiological studies on interactions between comorbidities and COPD	II	0.741	0.411	0.750	0.603	0.414	0.533	0.575	0.534
148	Exploring the approaches to personalize the treatment of patients with COPD based on physical, emotional and social treatable traits	V	0.613	0.532	0.581	0.629	0.468	0.629	0.575	0.505
149	Establishing criteria for response to different COPD treatments	III	0.707	0.500	0.724	0.621	0.345	0.552	0.575	0.520
150	Exploring if case-finding and evidence-based treatment for tobacco dependence can improve the outcomes of inhaler trials for COPD in smokers	III	0.707	0.552	0.672	0.552	0.379	0.583	0.574	0.495
151	Studying the real difference in systemic risk of fractures, adrenal suppression, cataracts and diabetes when comparing ICS containing triple or dual therapy with either fluticasone furoate, fluticasone propionate, budesonide or beclomethasone in GOLD D patients with COPD	III	0.806	0.450	0.767	0.667	0.350	0.400	0.573	0.578
152	Identifying tools to phenotype and sub-phenotype COPD patients into more homogeneus groups related to clinical presentation, prognosis or response to treatment	V	0.883	0.468	0.677	0.661	0.258	0.484	0.572	0.583
153	Studying the effectiveness of early treatment triggered by algorithms based on different predictive markers in improving COPD outcomes	III	0.707	0.500	0.714	0.589	0.379	0.533	0.571	0.510
154	Defining the interrelationship between phenotypes and endotypes in determining new therapeutic strategies	II	0.750	0.500	0.690	0.617	0.400	0.467	0.570	0.529
155	Exploring how do clinical features and biomarkers differ between COPD caused by different risk factors	I	0.773	0.438	0.766	0.790	0.281	0.375	0.570	0.603
156	Defining the optimal use for registries to address the challenges of over/under diagnosis of COPD in the real world	V	0.818	0.468	0.688	0.548	0.274	0.609	0.568	0.603

157	Exploring if shared decision-making can improve long-term health benefits in COPD	III	0.714	0.464	0.655	0.586	0.362	0.621	0.567	0.520
158	Understanding the impact and genesis of early COPD through critical assessment of early loss of lung function / rapid later loss of lung function	II	0.673	0.519	0.661	0.500	0.556	0.483	0.565	0.446
159	Identification of all treatable traits in COPD: pulmonary, extrapulmonary and environmental	III	0.600	0.552	0.583	0.569	0.517	0.567	0.565	0.461
160	Identifying the optimal strategies to identify and document progression of early disease - pre-COPD	V	0.661	0.534	0.690	0.431	0.397	0.667	0.563	0.510
161	Studying why are H. influenzae and S. pneumoniae colonisation and infection so specifically prevalent in COPD and whether this relates to upregulation of epithelial adhesion sites	II	0.833	0.463	0.815	0.482	0.357	0.429	0.563	0.520
162	Calibrating the duration of physical activity using pedometers required to change sedentary behaviour of COPD patients	IV	0.931	0.345	0.750	0.583	0.367	0.400	0.563	0.588
163	Identifying the needs of COPD patients at different stages of the disease	V	0.690	0.483	0.667	0.569	0.367	0.600	0.562	0.529
164	Studying the impact and progression of established and highly characterised COPD clinical phenotypes	II	0.865	0.423	0.732	0.574	0.288	0.481	0.561	0.500
165	Identifying feasible approaches to change movement behaviour of patients with COPD	III	0.661	0.393	0.759	0.483	0.414	0.633	0.557	0.510
166	Studying the real difference in pneumonia risk when comparing ICS containing triple or dual therapy with either fluticasone furoate, fluticasone propionate, budesonide or beclomethasone in GOLD D patients with COPD	III	0.800	0.397	0.897	0.724	0.241	0.276	0.556	0.632
167	Studying if physical activity in early life influences the development of COPD	I	0.683	0.567	0.563	0.452	0.484	0.581	0.555	0.520
168	Identifying the proportion of patients with COPD that receive evidence-based medications in different contexts	III	0.828	0.345	0.733	0.567	0.300	0.550	0.554	0.569

169	Exploring whether educational and quality improvement strategies promote registry robustness and patient care across COPD severity categories	III	0.768	0.414	0.667	0.517	0.433	0.517	0.553	0.515
170	Studying if cardio-selective beta-blockers (eg., bisoprolol) affect mortality in COPD patients without overt cardiovascular disease	III	0.750	0.481	0.714	0.607	0.315	0.446	0.552	0.515
171	Identifying clinical features that allow distinguishing acute exacerbations of COPD from community acquired pneumonia in low resource settings in the absence of chest X rays?	V	0.776	0.370	0.633	0.567	0.328	0.633	0.551	0.539
172	Studying if mucus clearance from the airway lumen is necessary to resolve symptoms and allow effective delivery of aerosol therapies in patients with mucus hypersecretion	III	0.768	0.464	0.724	0.534	0.321	0.483	0.549	0.505
173	Identifying molecular subtypes of COPD	II	0.875	0.375	0.793	0.552	0.250	0.448	0.549	0.574
174	Exploring the role of sarcopenia in COPD - its determinants, prognostic implications and potential interventions	II	0.804	0.339	0.750	0.600	0.276	0.517	0.548	0.569
175	Establishing subtypes of COPD using different aspects of the patient (eg., genome, microbiome, imaging techniques, pulmonary function test, exercise test, fat free mass / body composition), environment and deep learning - a step further than personalized medicine	II	0.810	0.467	0.694	0.552	0.317	0.433	0.545	0.544
176	Identifying molecular risk factors for disease progression in COPD	I	0.839	0.450	0.776	0.450	0.411	0.345	0.545	0.559
177	Studying the role of autoimmunity (B and/or T cells mediated) in progression of stable COPD	II	0.788	0.333	0.857	0.554	0.339	0.393	0.544	0.525
178	Developing and adopting COPD classification based on treatable traits	V	0.724	0.464	0.586	0.638	0.304	0.534	0.542	0.510
179	Developing techniques more rapid than spirometry to measure disease progression	III	0.667	0.500	0.565	0.661	0.339	0.516	0.541	0.515
180	Developing and validating multidimensional classification of COPD phenotypes	II	0.750	0.259	0.717	0.617	0.397	0.500	0.540	0.554

181	Exploring the role for immunotherapy in early stage COPD patients	III	0.685	0.554	0.655	0.500	0.379	0.448	0.537	0.490
182	Identifying approaches to combining domain knowledge from current COPD practice with the latest technological sensing technology (both objective and subjective) and data science methods, to tailor care to the individual patient (e.g. determining the optimal referral strategy, contents of rehabilitation programs, developing highly-engaging PA coaching programs for home, etc.)	V	0.655	0.448	0.517	0.467	0.567	0.567	0.537	0.461
183	Studying and comparing the impact of environmental factors (eg., air pollutants, infections and occupational dust) on lung inflammation and discovery of novel therapeutic targets	I	0.638	0.534	0.567	0.500	0.379	0.600	0.536	0.480
184	Studying the impact of valsartan/sacubitril (Entresto) on morbidity and mortality in patients with COPD who have pulmonary heart disease (cor pulmonale) with or without LTOT	III	0.839	0.352	0.810	0.643	0.172	0.397	0.536	0.583
185	Investigate the cost-effectiveness of screening of DAAT in COPD patients	V	0.827	0.423	0.780	0.500	0.208	0.462	0.533	0.490
186	Studying patient perceptions of the reasons they are admitted to hospital with an acute exacerbation of COPD	IV	0.726	0.339	0.790	0.563	0.297	0.469	0.530	0.598
187	Studying patients' perspective and perceptions of the disease and their expectation about diagnosis and management of the disease	IV	0.790	0.233	0.703	0.500	0.406	0.531	0.527	0.593
188	Explaining the different frequency of exacerbations among persons with the same degree of airway obstruction	II	0.759	0.411	0.650	0.414	0.345	0.567	0.524	0.534
189	Studying the role of parental health in the development of COPD	I	0.667	0.531	0.672	0.387	0.297	0.563	0.519	0.578
190	Studying COPD with all comorbidities to better understand the underlying shared biological mechanisms to target new or repurpose existing therapeutic targets	III	0.583	0.517	0.597	0.517	0.355	0.532	0.517	0.480

191	Identifying pathogenic mechanism involved in the development of the different subtypes of COPD (genetic, immunological, endocrinologcal and metabolic)	II	0.696	0.463	0.517	0.554	0.375	0.483	0.515	0.461
192	Exploring if measuring lung stiffness as reactance area (AX) using impulse oscillometry better identifies early COPD	V	0.889	0.385	0.768	0.463	0.250	0.310	0.511	0.559
193	Studying if there are common molecular pathways that mediate the increased risk of lung carcinoma (both NSCLC and SCLC) in COPD patients	II	0.850	0.344	0.767	0.400	0.344	0.359	0.511	0.618
194	Analysis of genome-wide association studies for COPD to eludicate specific biological pathways	II	0.759	0.429	0.732	0.464	0.321	0.357	0.510	0.510
195	Exploring if lung clearance index and airway oscillometry can predict COPD	V	0.759	0.340	0.696	0.500	0.389	0.357	0.507	0.500
196	Developing early risk profiling for COPD based on a poor early adult lung function due to childhood insults, active asthma into middle age and reactive oxygen species airway cellular activation and damage from smoking and air pollution	I	0.483	0.655	0.534	0.375	0.534	0.448	0.505	0.466
197	Studying if a multi-dimensional approach and advanced machine-learning techniques may allow a clinically meaningful phenotyping of patients with COPD	V	0.815	0.250	0.621	0.500	0.328	0.517	0.505	0.529
198	Studying of therapies that reduce airway glucose (eg. metformin) are effective in reducing bacterial infections in COPD	III	0.759	0.407	0.714	0.482	0.250	0.414	0.504	0.525
199	Determining the importance and biomarker potential of innate and adaptive immune system responses and relevance to disease exacerbations and progression.	V	0.732	0.339	0.611	0.554	0.357	0.414	0.501	0.505
200	Exploring relationships between enviromental factors and multilevel -omics in COPD, including genomics, epigenomics, transcriptomics, proteinomics and metabolomics.	I	0.654	0.481	0.696	0.414	0.321	0.429	0.499	0.480

201	Studying what determinants at an early stage of the disease condition the final COPD phenotypes (eg., emphysema, chronic bronchitis, “pink puffer", “blue bloater”)	I	0.750	0.393	0.569	0.357	0.414	0.483	0.494	0.495
202	Understanding endothelial nitric oxide pathway and the association with airway inflammation in COPD	II	0.804	0.393	0.672	0.389	0.268	0.414	0.490	0.539
203	Describing the characteristics of airway remodelling in COPD	II	0.810	0.286	0.776	0.466	0.224	0.367	0.488	0.588
204	Identifying common molecular pathways triggering acute on chronic lower airways inflammation during COPD exacerbations of different levels of severity	II	0.732	0.389	0.589	0.463	0.375	0.379	0.488	0.495
205	Understanding the relationship between endotype and phenotype Identifying trigger mechanisms for COPD in predisposed individuals	II	0.680	0.466	0.554	0.500	0.328	0.379	0.484	0.466
206	Assessing the risk effect of 1-year history of mild and moderate ambulatory exacerbations on mortality in patients with COPD	VI	0.750	0.367	0.883	0.304	0.217	0.367	0.481	0.618
207	Studying the cause of impairment of the cellular and molecular mechanisms of lower airways regeneration in COPD patients	II	0.660	0.462	0.538	0.462	0.288	0.464	0.479	0.436
208	Agreeing on best research design ‘beyond RCT’ to investigate the effectiveness of rapidly advancing and changing e-health and m-health innovations in COPD	III	0.586	0.400	0.583	0.484	0.422	0.375	0.475	0.520
209	Studying how different types of gastro-oesophageal reflux associate with risk of COPD exacerbations and exploring optimal treatment options	I	0.800	0.328	0.621	0.431	0.259	0.379	0.470	0.559
210	Investigating the reasons of variability of the effect of Alpha1 antitrypsin deficiency	II	0.808	0.340	0.558	0.500	0.204	0.393	0.467	0.495
211	Validating of objective and subjective clinical outcomes specific for disease modifying therapies	III	0.558	0.346	0.589	0.519	0.259	0.500	0.462	0.461

212	Identification of non-coding RNAs that contribute to COPD pathophysiology and development of inhalable ncRNA-based medicines for treatment of COPD symptomology	II	0.712	0.385	0.667	0.389	0.278	0.321	0.458	0.510
213	Studying value-based COPD care and identifying important values for patients and society to achieve in COPD care (in line with Machteld Huber's 'positive health').	III	0.574	0.370	0.519	0.429	0.224	0.621	0.456	0.495
214	Identifying drivers of epithelial-mesenchymal transition (EMT) in smoking-related COPD and its relation to the severe risk of COPD and airway cancers	I	0.732	0.389	0.643	0.407	0.241	0.321	0.456	0.525
215	Studying the early effects causing a lower maximum lung function at young adulthood and its clinical implications	VI	0.558	0.446	0.625	0.352	0.304	0.429	0.452	0.480
216	Identifying biological pathways that underlie different clinical presentations	II	0.635	0.310	0.500	0.481	0.250	0.500	0.446	0.461
217	Exploring if COPD with airway mucus hypersecretion and higher risk of dying should be approached as a separate disease	II	0.565	0.328	0.583	0.533	0.300	0.367	0.446	0.525
218	Exploring the basis of day-to-day variability in symptoms (eg., dyspnoea, fatigue, pain, weakness, insomnia, guilt, anxiety, depression, appetite, etc.) in patients with COPD	VI	0.685	0.259	0.625	0.389	0.179	0.483	0.437	0.525
219	Exploring if measurement of lung stiffness as reactance area (AX) using impulse oscillometry can identify treatment response to LABA/LAMA or triple therapy in patients with GOLD B/D	III	0.793	0.222	0.707	0.464	0.154	0.259	0.433	0.569
220	Identifying diagnostic tools to distinguish between the true COPD exacerbation and just a 'bad day'	V	0.569	0.323	0.583	0.552	0.155	0.367	0.425	0.544
221	Studying if chronic non-fully reversible airflow obstruction in never-smokers is really COPD	III	0.630	0.286	0.569	0.339	0.241	0.483	0.425	0.515
222	Developing new models of care in COPD based on the coming "silver tsunami" and limited resources	III	0.500	0.308	0.407	0.407	0.385	0.500	0.418	0.426

223	Studying the pathophysiological processes that lead to emphysema, bronchiectasis and chronic bronchitis - are they the same but just affecting different anatomical sites?	II	0.630	0.321	0.500	0.321	0.286	0.379	0.406	0.515
224	Understanding the basic non-hypoxic pathogenesis of pulmonary hypertension in COPD	II	0.667	0.241	0.607	0.431	0.103	0.379	0.405	0.564
225	Studying if adverse effects of beta agonists, taken in the absence of inhaled corticosteroids, are an unrecognised problem in COPD	III	0.692	0.167	0.625	0.393	0.148	0.393	0.403	0.554
226	Studying gene reprogramming of the epithelium in COPD	II	0.560	0.280	0.558	0.288	0.120	0.357	0.361	0.495
227	Piloting replacement of diagnosis "COPD" with individual clinical and biological phenotyping	V	0.571	0.293	0.500	0.310	0.172	0.310	0.360	0.554
228	Evaluating the usefulness of measuring sensory and affective dimension of acute or episodic breathlessness (clinical, spirometry, EEG and fMRI) to differentiate breathlessness from panic in COPD	VI	0.556	0.214	0.500	0.414	0.155	0.300	0.356	0.554
229	Evaluating animal models to determine the contributions of new generation of nicotine products	III	0.556	0.308	0.482	0.315	0.125	0.310	0.349	0.525
230	Synthesizing various lines of evidence to reach a consensus whether COPD is a disease or a disorder	V	0.365	0.111	0.278	0.259	0.111	0.333	0.243	0.593

Table S8: Contributors to the Global COPD CHNRI exercise.

Name	Country	Region	Income
1. Agarwal, Dhiraj	India	South-East Asia	LMIC
2. Barnes, Peter J	UK	Europe	HIC
3. BONAY, Marcel	France	Europe	HIC
4. Boven, van, J.F.M.	Netherlands	Europe	HIC
5. Brooks, Dina	Canada	Americas	HIC
6. Bryant, Jamie	Australia	Western Pacific	HIC
7. Campbell, Harry	UK	Europe	HIC
8. Caramori, Gaetano	Italy	Europe	HIC
9. Cazzola, Mario	Italy	Europe	HIC
10. Cho, Michael	USA	Europe	HIC
11. Cristóbal Esteban	Spain	Europe	HIC
12. Divo, Miguel	USA	Europe	HIC
13. Dockrell, David	UK	Europe	HIC
14. D'Urzo, Anthony D.	Canada	Americas	HIC
15. Ekström, Magnus	Sweden	Europe	HIC
16. Erharbor, Gregory	Nigeria	Africa	LMIC
17. Feldman, Gregory	USA	Americas	HIC
18. Fonseca, João A	Portugal	Europe	HIC
19. Gemert, Frederik	Netherlands	Europe	HIC
20. Greene, Catherine	Ireland	Europe	HIC
21. Hall, Ian	UK	Europe	HIC
22. Hurst, John	UK	Europe	HIC
23. Johnston, Sebastian L	UK	Europe	HIC
24. Juvekar, Sanjay	India	South-East Asia	LMIC
25. Kankaanranta, Hannu	Finland	Europe	HIC
26. Khoo, E E Ming	Malaysia	Western Pacific	LMIC
27. Ko, Fanny Wai San	Hong Kong, China	Western Pacific	LMIC
28. Lahousse, Lies	Belgium	Europe	HIC
29. Lindenauer, Peter	USA	Americas	HIC
30. Lipworth, Brian	UK	Europe	HIC
31. Lopez-Campos, Jose Luis	Spain	Europe	HIC
32. Maddocks, Matthew	UK	Europe	HIC
33. Mannino, David	USA	Americas	HIC
34. Martinez Garcia, Miguel Angel	Spain	Europe	HIC
35. Martinez, Fernando J.	USA	Americas	HIC
36. McCarthy, Bernard	Ireland	Europe	HIC
37. Mcnamara, Renae	Australia	Western Pacific	HIC
38. Mendoza, Laura	Chile	Americas	LMIC
39. Miravitlles, Marc	Spain	Europe	HIC
40. Pedone, Claudio	Italy	Europe	HIC
41. Pinnock, Hilary	UK	Europe	HIC
42. Pooler, Alison	UK	Europe	HIC
43. Quint, Jennifer K.	UK	Europe	HIC
44. Reddy, Raju	USA	Americas	HIC

45. Sadatsafavi, Mohsen	USA	Americas	HIC
46. Schwarz, Peter	Denmark	Europe	HIC
47. Simon, Steffen	Germany	Europe	HIC
48. Smith, Benjamin M.	USA	Americas	HIC
49. Soriano, Joan B.	Spain	Europe	HIC
50. Spruit, Martijn A.	Netherlands	Europe	HIC
51. Sterk, P.J.	Netherlands	Europe	HIC
52. Stockley, Rob	UK	Europe	HIC
53. Tabak, Monique	Netherlands	Europe	HIC
54. Tai, Andrew	Australia	Western Pacific	HIC
55. Thanavala, Yasmin	USA	Americas	HIC
56. van der Eerden, Menno, M.	Netherlands	Europe	HIC
57. Vestbo , Jorgen	UK	Europe	HIC
58. Walters, Haydn	Australia	Western Pacific	HIC
59. Wark, Peter	Australia	Western Pacific	HIC
60. Watz, Henrik	Germany	Europe	HIC
61. Wedzicha, Jadwiga A.	UK	Europe	HIC
62. Williams, Michelle	UK	Europe	HIC
63. Williams, Sian	UK	Europe	HIC
64. Yusuf, Osman	Pakistan	South-East Asia	LMIC
65. Adeloye, Davies	Nigeria	Africa	LMIC
66. Rudan, Igor	UK	Europe	HIC

SUPPLEMENTARY FIGURES

Figure S1. Flow chart of the COPD CHNRI research prioritization exercise

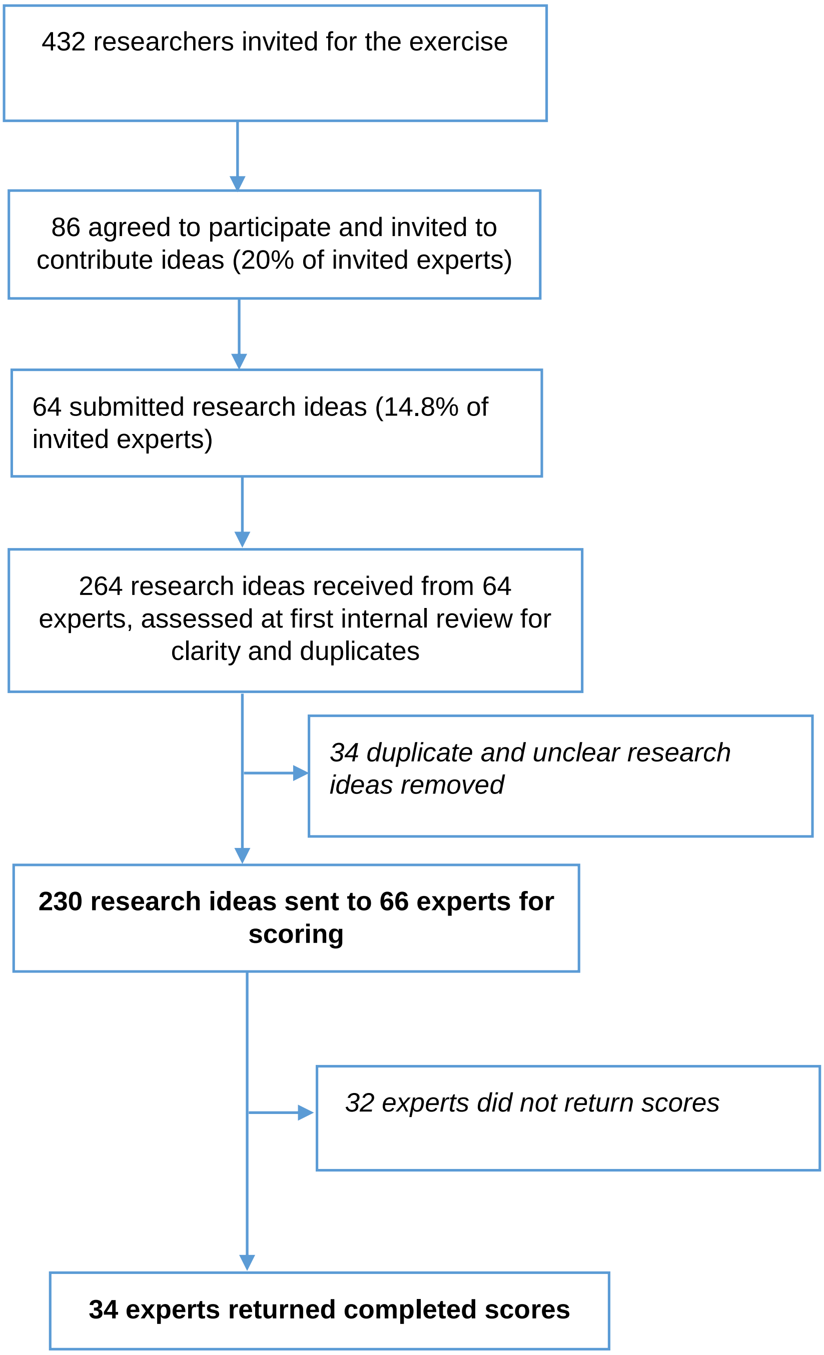

Supplement: Online Supplementary Document [file jogh-11-15003-s001.pdf]
